# Hippocampal perineuronal net degradation identifies prefrontal and striatal circuits involved in schizophrenia-like changes in marmosets

**DOI:** 10.1126/sciadv.adu0975

**Published:** 2025-04-18

**Authors:** Miriam A. Gwilt, Amy R. Hodgson, Sebastian F. A. Axelsson, Gemma J. Cockcroft, Lauren B. McIver, Matthew Hird, Arkadiusz Stasiak, Colin McKenzie, Samantha H.-Y. Ip, Rudolf N. Cardinal, Stephen J. Sawiak, Selena Milicevic Sephton, Franklin I. Aigbirhio, Young T. Hong, Timothy D. Fryer, Hannah F. Clarke

**Affiliations:** ^1^ Department of Physiology, Development and Neuroscience, University of Cambridge, Cambridge, UK.; ^2^ Department of Psychology, University of Cambridge, Cambridge, UK.; ^3^ Molecular Imaging Chemistry Laboratory, Cambridge Biomedical Campus, West Forvie building, Robinson Way, CB2 0SZ Cambridge, UK.; ^4^ British Heart Foundation Cardiovascular Epidemiology Unit, Department of Public Health and Primary Care, University of Cambridge, Cambridge, UK.; ^5^ Cambridge Centre for AI in Medicine, Department of Applied Mathematics and Theoretical Physics, University of Cambridge, Cambridge, UK.; ^6^ Department of Psychiatry, University of Cambridge, and Cambridgeshire and Peterborough NHS Foundation Trust, Cambridge, UK.; ^7^ Wolfson Brain Imaging Centre, University of Cambridge, Cambridge, UK.

## Abstract

In schizophrenia, anterior hippocampus (aHipp) overactivity is associated with orbitofrontal cortex (OFC) dysfunction, but the contribution to symptomatology is unknown. In rodents, degradation of the hippocampal perineuronal net (PNN) replicates this overactivity, but uncertainty over rodent/human prefrontal homology limits translation to humans. Here, we test the hypothesis that aHipp PNN degradation in a species with a human-like prefrontal cortex, the marmoset, alters aHipp-striatal and aHipp-OFC circuitry. Microdialysis and [^18^F]-fluoro-l-dihydroxyphenylalanine positron emission tomography identified increased dopamine synthesis in the associative striatum, but not the nucleus accumbens, as is seen in schizophrenia, and elevated dopamine and noradrenaline in the OFC. Behaviorally, activity was elevated in a marmoset version of the amphetamine-induced activity test, and impaired probabilistic discrimination learning was seen in an OFC/striatum-dependent task that computational modeling suggests was due to loss of goal-directed behavior. Together, these findings demonstrate that a loss of primate aHipp PNNs is sufficient to induce striatal and prefrontal dysfunction consistent with that observed in humans with schizophrenia.

## Teaser

Marmoset hippocampal perineuronal net loss causes schizophrenia-like changes in striatal and orbitofrontal function.

## INTRODUCTION

Over the past few decades, it has become increasingly apparent that abnormalities of the hippocampus are fundamental to the pathophysiology of schizophrenia (*1*). Hippocampal overactivity progresses during the development of the disorder (*2*, *3*) and is accompanied by a decrease in hippocampal volume. The degree of hippocampal overactivity is strongly correlated with the severity of the “positive” (psychotic) symptoms of schizophrenia, and hippocampal overactivity is observed in many rodent models of psychosis, where it has been shown to underpin schizophrenia-related deficits including impaired sensorimotor gating and disinhibition of striatal dopamine signaling.

While the positive symptoms of schizophrenia (the delusions and hallucinations) are often the most notable, schizophrenia is also associated with profound cognitive impairments and “negative”/emotional symptoms such as behavioral inflexibility, anhedonia, and social withdrawal. Unlike the positive symptoms, which are strongly associated with excess striatal dopamine and are therapeutically targeted by dopamine D2 receptor antagonist antipsychotics, these cognitive/negative symptoms may be mediated by different pathophysiological processes (*4*), are poorly treated by current medications, profoundly affect the quality of life, and represent an urgent unmet clinical need (*5*, *6*).

Schizophrenia-related hippocampal overactivity is particularly prominent in the anterior hippocampus (aHipp) (*2*, *7*, *8*), the hippocampal region most strongly connected with the limbic and frontal structures thought to be critical to cognitive and emotional functions. Functional neuroimaging studies in patients with schizophrenia reveal reduced activation in the dorsolateral prefrontal cortex, anterior cingulate cortex, and thalamus, as well as a progressive increase in orbitofrontal cortex (OFC) activity that is correlated with the increasing hippocampal activity seen during the early development of schizophrenia (*2*, *3*, *9*). However, such studies do not demonstrate causality, allowing the possibility of reverse and third-factor causality. Determining any causal relationship between aHipp overactivity and prefrontal/cingulate dysfunction is therefore fundamental to the development of novel treatments for these symptoms.

In the rodent, enzymatic degradation of the hippocampal perineuronal net (PNN), a component of the extracellular matrix, successfully recapitulates the hippocampal overactivity seen in schizophrenia and is accompanied by striatal dopaminergic disinhibition, suggesting that this approach has utility for investigating schizophrenia-related pathology (*10*). However, translation from rodent studies is limited by uncertainty over the homology between rodent and human prefrontal cortices, and there is also uncertainty regarding the location of the striatal dopamine changes. In rodent studies, striatal dopamine changes are quantified by the increased dopamine in the nucleus accumbens (Acb) and via the behavioral hyperactivity seen after treatment with low doses of psychostimulants such as amphetamine (*10*–*13*). However, meta-analyses of imaging techniques in humans, such as [^18^F]-fluoro-l-dihydroxyphenylalanine ([^18^F]-DOPA) positron emission tomography (PET) imaging of dopamine synthesis, identify changes within associative striatal structures and not the Acb (*14*, *15*). We therefore sought to determine whether aHipp PNN degradation in a marmoset monkey—a species with a human-like structure to its striatum and prefrontal cortex—would induce alterations in striatal and prefrontal function and neurochemistry relevant to schizophrenia symptoms and its treatment.

In this study, we developed aHipp PNN degradation in the marmoset monkey and interrogated its effects on aHipp-striatal and aHipp-OFC circuitry. We used in vivo microdialysis and [^18^F]-DOPA PET to quantify resulting alterations in striatal catecholamine levels. We assessed behavior known to depend on striatal catecholamines using a novel marmoset version of the amphetamine-induced hyperlocomotion task. We then extended this approach into the prefrontal cortex and focused on the OFC because of its correlation with hippocampal activity in schizophrenia (*16*). OFC microdialysis revealed altered catecholamine function that was accompanied by impaired probabilistic discrimination learning, known to depend on OFC-striatal circuitry, with alterations in goal-directed processes suggested by computational modeling. Together, these experiments reveal that PNN degradation in the primate aHipp alters behaviors that are relevant to the cognitive symptoms of schizophrenia and reveals the alterations in OFC neurochemistry that underpin them.

## RESULTS

### aHipp PNN degradation increases stereotypical behavior and striatal dopamine synthesis

**Fig. 1.**
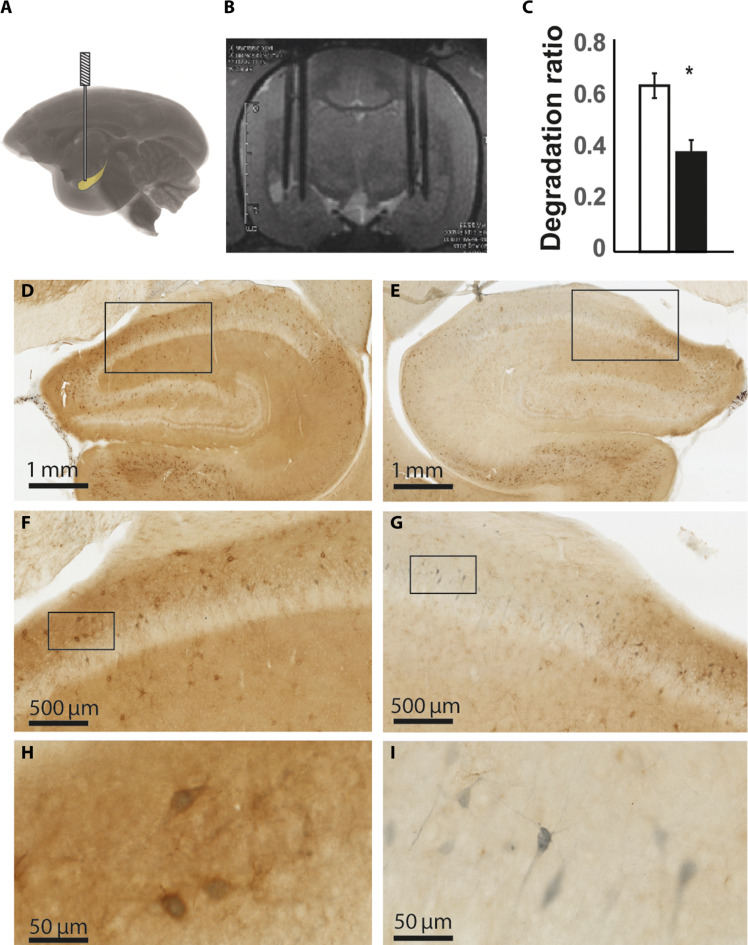
PNNs were selectively degraded throughout the aHipp. (**A**) Schematic depicting bilateral cannulae targeting the aHipp. (**B**) Representative MRI of bilateral double cannulae targeting the aHipp. (**C**) Quantification of PNN staining after unilateral chondroitinase ABC digestion. The ratio of PNN staining in the degraded hippocampus (black) as a fraction of all staining is reduced when compared to the intact hippocampus (white). Means ± SEM; *P < 0.05. (**D** to **I**) PNNs (brown) surround PV-positive interneurons (gray) throughout the aHipp. Photomicrographs at increasing levels of magnification (from top to bottom) in an intact [(D), (F), and (H)] and degraded [(E), (G), and (I)] hippocampus demonstrate the loss of PNNs in the latter. In each case, the area enclosed in the rectangle is enlarged in the image immediately below. Panels (H) and (I) specifically demonstrate the loss of PNNs from around PV-positive cells.

Marmosets were implanted with bilateral cannulae targeting the aHipp, which permitted the infusion of the enzyme chondroitinase ABC from *Proteus vulgaris* [or 0.1% bovine serum albumin (BSA) vehicle] specifically into the aHipp (*10*) (Fig. 1, A and B). Chondroitinase ABC digests glycosaminoglycans including chondroitin sulfate and hyaluronan, which are key components of the PNN, and therefore disrupts its structure. Postmortem histological analysis revealed that chondroitinase ABC treatment resulted in a robust decrease in the amount of PNN staining that was anatomically localized to the aHipp (*t*_5_ = −2.688, *P* = 0.043; Fig. 1C and fig. S1).

To determine how marmoset aHipp PNN degradation influenced striatal dopamine, we combined approaches from the rodent and human literature. Thus, we developed a home cage–based marmoset version of the amphetamine-induced activity task used in rodents, alongside in vivo microdialysis of the Acb, and we performed [^18^F]-DOPA PET imaging to understand how dopamine synthesis changed across the whole striatum, as in humans (*14*).

**Fig. 2.**
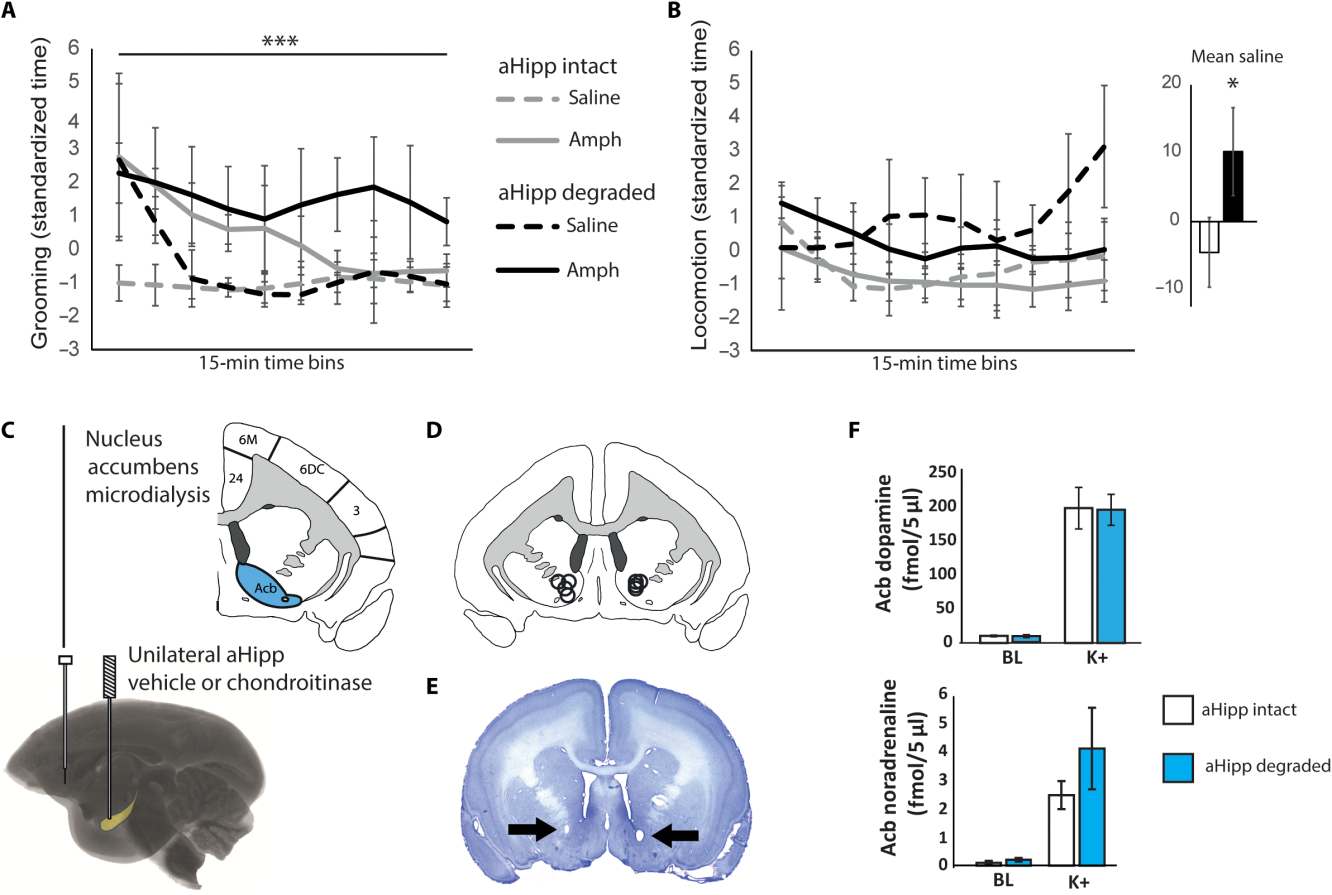
aHipp PNN degradation increases activity but does not alter extracellular dopamine levels in the Acb. (**A**) Bilateral aHipp PNN degradation amplified the stereotypical grooming behavior observed after intramuscular amphetamine (0.5 mg/kg; *n* = 4) treatment; ***P < 0.001. Means ± SEM. (**B**) Amphetamine did not affect locomotor activity, but aHipp PNN degradation alone was sufficient to increase the mean locomotor activity observed after saline injection across all time bins. Inset: mean locomotor activity before (white bar) and after (black bar) PNN degradation and saline treatment (*n* = 4); *P < 0.05. Means ± SEM. (**C**) Schematic depicting the experimental Acb microdialysis procedure. (**D**) Schematic depicting the location of the dialysis probes within the Acb of each animal, as determined by postmortem histology, plotted here on a single coronal section (AP 11 to 12.2). For histological schematics of the dialysis subjects, see fig. S2. (**E**) Representative histological section, with arrows marking the small holes at the position of the cannula tips. (**F**) Extracellular dopamine and noradrenaline levels within the Acb under tonic baseline (BL) conditions and phasic conditions (evoked by 75 mM K^+^; K+) were unchanged by aHipp PNN degradation (*n* = 4; means ± SEM).

We compared the effects of intramuscular amphetamine (0.5 mg/kg) versus saline (factor “drug”) treatment on stereotypical self-grooming and locomotor activity after both aHipp vehicle treatment and aHipp PNN degradation (factor “degradation”). Consistent with the increase in activity seen with amphetamine in rodents, marmosets treated with aHipp chondroitinase ABC showed a substantial increase in self-grooming compared to aHipp vehicle treatment [Fig. 2A; repeated-measures analysis of variance (ANOVA), degradation × drug × time: *F*_9,27_ = 4.662, *P* < 0.001, *n* = 4]. However, this effect was also seen with saline treatment alone, indicating that while the amphetamine effect was larger, aHipp chondroitinase treatment alone was sufficient to increase self-grooming in response to the stimulation of a saline injection (degradation × time: *F*_9,27_ = 5.817, *P* < 0.001). Degradation also caused increased locomotion, but there was no effect of amphetamine (Fig. 2B; degradation: *F*_1,3_ = 28.98, *P* = 0.013; saline only, main effect of degradation: *F*_1,3_ = 12.807, *P* = 0.037).

To determine whether the increases in locomotion and grooming were related to increased dopamine release in the Acb, we measured extracellular catecholamine activity by implanting temporary microdialysis probes bilaterally into the Acb core and shell and performing in vivo microdialysis after unilateral aHipp PNN degradation (Fig. 2, C to E). We found no differences in either tonic/baseline or K^+^-evoked dopamine efflux in the Acb [Fig. 2F; tonic: intact (not degraded), 8.45 ± 0.78 fmol/5 μl (means ± SEM); degraded, 6.82 ± 1.67 fmol/5 μl, not significant (NS); K^+^-evoked: intact, 193.5 ± 23.9 fmol/5 μl; degraded, 191.1 ± 22.14 fmol/5 μl, NS; *n* = 4]. Noradrenaline was also unchanged (Fig. 2F; tonic: intact, 0.096 ± 0.065 fmol/5 μl; degraded, 0.205 ± 0.066 fmol/5 μl, NS; K^+^-evoked: intact, 2.47 ± 0.08 fmol/5 μl; degraded, 4.1 ± 1.43 fmol/5 μl, NS; *n* = 4). This suggests that the increases in activity observed were not due to increased dopamine release in the Acb but may have been due to dopaminergic changes elsewhere in the striatum, as seen in humans with schizophrenia.

**Fig. 3.**
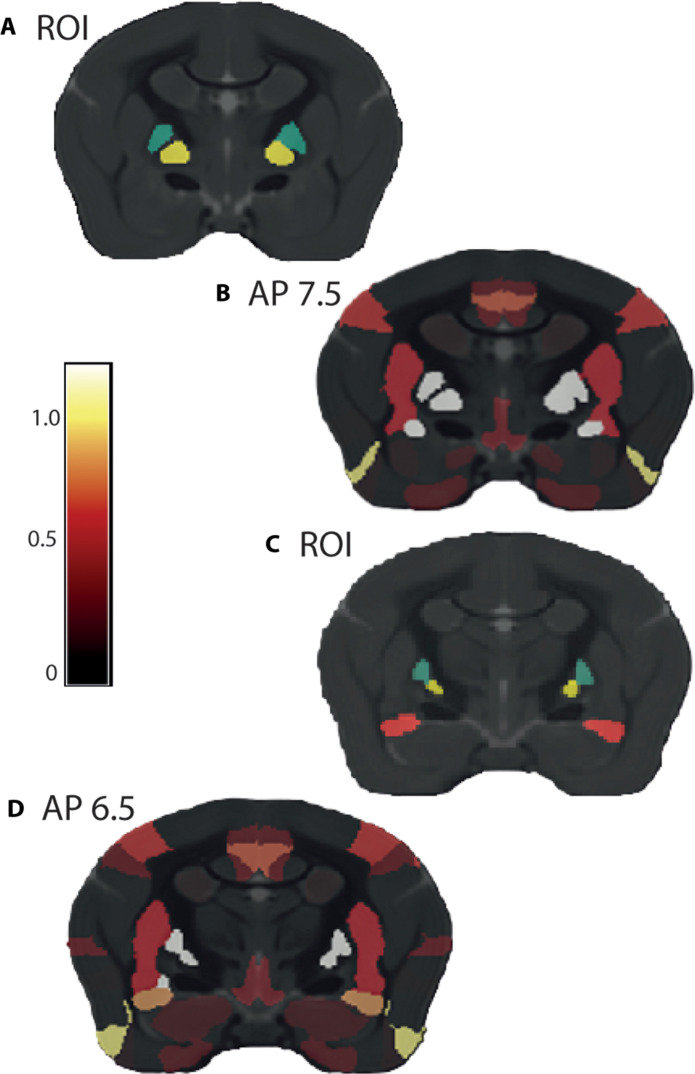
aHipp PNN degradation increases dopamine synthesis in the associative striatum. (**B** and **D**) Coronal co-registered MRI and −log_10_(*P* value) maps depicting the significance of regional increases in *k*_3_^s^, the rate at which [^18^F]-DOPA is decarboxylated to [^18^F]-fluorodopamine. Depicted at AP 7.5 (B) and AP 6.5 (D) alongside matched region of interest schematics (**A** and **C**), respectively (green, GPexternal; yellow, GPinternal; red, caudate tail). Data are analyzed by repeated-measures ANOVA (factors: scan_2_ × area_5_) with a priori striatal ROIs (anterior caudate, caudate tail, GPinternal, GPexternal, and putamen); scan × area, *P* = 0.036. Post hoc paired *t* tests localized the changes to the caudate tail (*P* = 0.02) and the GP (GPexternal, *P* = 0.015; GPinternal, *P* = 0.006) but not the putamen (*P* = 0.464) and the anterior caudate (*P* = 0.956) (*n* = 3). These changes were not due to PNN degradation within either structure (see fig. S7). aHipp PNN degradation also increased dopamine synthesis within the central nucleus of the amygdala. The scale bar has an upper limit of 1.3, which corresponds to *P* = 0.05. For histological schematics of the PET-scanned and locomotion subjects, see fig. S3. For relative *k*_3_^s^ ratios and raw striatal *k*_3_^s^ numbers, see tables S4 and S5.

Using [^18^F]-DOPA PET imaging, we next assessed dopamine synthesis across the whole striatum. Comparison within the basal ganglia before and after bilateral aHipp PNN degradation revealed selective regional changes in dopamine synthesis within associative striatal structures including the caudate tail (paired *t* test, *t*_2_ = −7.0, *P* = 0.02, *n* = 3) and the globus pallidus (GP; GPexternal, *t*_2_ = −8.0, *P* = 0.015; GPinternal, *t*_2_ = −13.0, *P* = 0.006, *n* = 3), a structure with limbic and associative subdivisions (*17*). No changes were seen in the putamen or anterior caudate regions, indicating that aHipp PNN degradation in a monkey produces a distribution pattern of striatal hyperdopaminergia more representative of human schizophrenia. Dopamine synthesis was also elevated in the amygdala, as is seen clinically (*18*). Representative images are shown in Fig. 3. Together, these results provide a causal demonstration of the effects of aHipp PNN degradation in the primate, bridge the explanatory gap between rodent causal experiments and human correlative studies, and demonstrate the utility of primate aHipp PNN degradation for investigating schizophrenia-related pathology.

### aHipp PNN degradation alters orbitofrontal neurochemistry and behavior

**Fig. 4.**
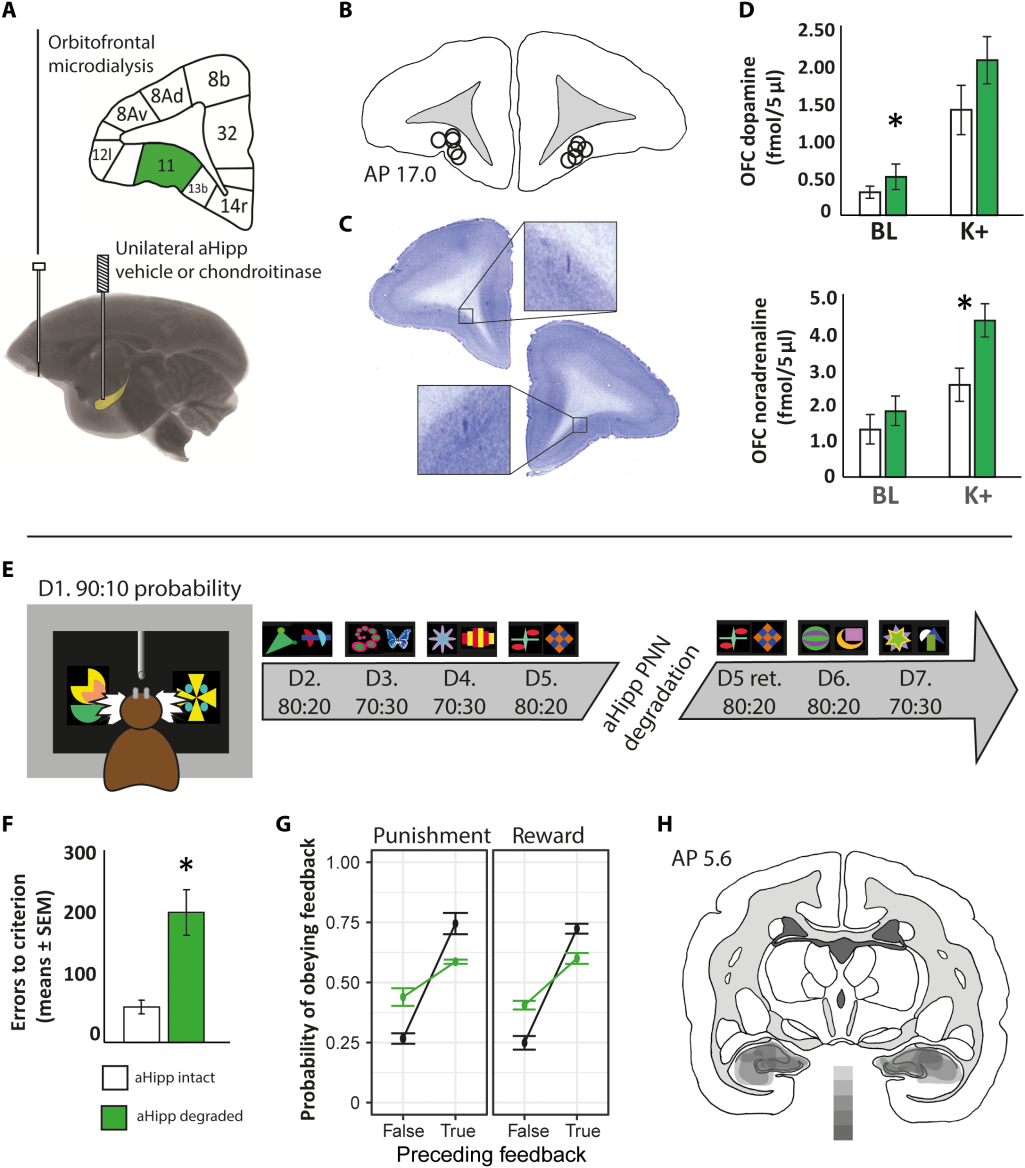
aHipp PNN degradation alters orbitofrontal neurochemistry and behavior. (**A**) Marmosets were implanted with chronic bilateral cannulae targeting the aHipp to permit unilateral infusion of chondroitinase ABC in the left hemisphere (while the right received vehicle). Dialysis probes were then implanted acutely into the OFC bilaterally. (**B**) Location of the dialysis probes within the OFC of each animal, plotted here on a single coronal section (AP 15.8 to 17). (**C**) Representative histological sections, with enlarged areas highlighting the position of the cannula tips. (**D**) Extracellular baseline (BL) dopamine and phasic (K^+^) noradrenaline levels within the OFC were increased by aHipp PNN degradation (*n* = 5; means ± SEM). *P < 0.05. (**E**) Behavioral visual discrimination task sequence (D*n*, discrimination number *n*). Representative stimuli are shown. In each case, one stimulus was correct and one incorrect, counterbalanced between individuals. Reinforcement probabilities are shown: For example, “90:10 probability” indicates that *P*(reward|correct stimulus selected) = *P*(punishment|incorrect stimulus selected) = 0.9 (majority or “true” feedback) and *P*(punishment|correct stimulus selected) = *P*(reward|incorrect stimulus selected) = 0.1 (minority or “false” feedback). (**F**) aHipp PNN degradation impaired probabilistic visual discrimination learning, with more errors to criterion compared to predegradation performance (*n* = 5). D5 retention was unaffected (NS). *P < 0.05. (**G**) aHipp PNN degradation impaired reward- and punishment-related behaviors. Degraded subjects showed a decreased probability of responding appropriately to the true feedback and an increased probability of responding to the misleading (false) feedback. Means ± SEM. *P < 0.05. (**H**) Schematic diagram showing the extent of the bilateral aHipp degradation in the discrimination monkeys (*n* = 5). From dark to light, the five shades of gray indicate the regions degraded in all five monkeys, any four monkeys, any three monkeys, any two monkeys, and one monkey, respectively.

Next, we aimed to determine whether altered OFC function, as seen in schizophrenia, could also be caused by aHipp PNN degradation. Consequently, we measured OFC extracellular neurotransmitter levels by implanting temporary microdialysis probes bilaterally into the OFC and performing in vivo microdialysis as before (Fig. 4, A to C).

This demonstrated increases in K^+^-evoked OFC noradrenaline release (Fig. 4D; baseline: intact, 1.24 ± 0.32 fmol/5 μl; degraded, 1.76 ± 0.39 fmol/5 μl, NS; K^+^-evoked: intact, 2.35 ± 0.42 fmol/5 μl; degraded, 4.01 ± 0.42 fmol/5 μl; *t*_4_ = −3.241, *P* = 0.032; *n* = 4) and increases in baseline but not K^+^-evoked OFC dopamine (baseline: intact, 0.28 ± 0.09 fmol/5 μl; degraded, 0.45 ± 0.11 fmol/5 μl; *t*_4_ = 4.707, *P* = 0.009; K^+^-evoked: intact, 1.39 ± 0.32 fmol/5 μl; degraded, 2.04 ± 0.31 fmol/5 μl; *t*_4_ = −2.180, *P* = 0.095; *n* = 4). Serotonin and glutamate were unchanged (see fig. S4). Thus, aHipp PNN degradation altered the neurochemical profile of the OFC in favor of elevated catecholamines.

To determine whether these OFC neurochemical changes, induced by aHipp PNN degradation, were associated with functional consequences, we tested cognition on a probabilistic discrimination learning task that is known to depend upon OFC/striatal dopamine in marmosets (*19*) and is impaired in schizophrenia (*20*). A new cohort of marmosets that received cannulae into the aHipp was presented with a series of two-choice probabilistic discriminations (90%:10%, 80%:20%, and 70%:30%) on a touch-sensitive computer screen, one stimulus of which was predominantly paired with reward (5-s banana milkshake) that the animal should learn to choose and the other stimulus predominantly paired with punishment (0.3-s, 100-dB loud noise) that the animal should learn to avoid. Therefore, animals not only needed to respond appropriately to the “true” (majority, i.e., 80%) feedback that accurately reflected their stimulus choice but also ignore the “misleading” (minority feedback, i.e., 20%) feedback. A discrimination was considered learnt when a criterion of more than 90% correct choices was made in one session. Their predegradation performance was then compared to their postdegradation performance (see Fig. 4E).

Animals with aHipp degradation were slower to learn new probabilistic discriminations, making more errors before reaching criterion performance, compared to their predegradation performance (mean errors to criterion during postdegradation discriminations versus predegradation discriminations, *t*_4_ = −4.713, *P* = 0.009, Fig. 4F). A preliminary analysis of behavior established that no term involving the reinforcement probabilities (90%:10%, 80%:20%, and 70%:30%) was significant (*F* ≤ 2.69, *P* ≥ 0.072), so performance was averaged across all probabilities. The data were then analyzed by repeated-measures ANOVA with factors of degradation (before and after), valence (correct and incorrect), and veracity (true and false/misleading).

Overall, degradation caused a broad impairment in learning that altered subjects’ ability to respond appropriately to “true” (majority) and “false/misleading” (minority) feedback after both rewarding and punishing trials (degradation × veracity, *F*_1,32_ = 60.6, *P* < 0.0001; veracity, *F*_1,32_ = 160.6, *P* < 0.0001; valence, *F*_1,32_ = 0.29, NS; *n* = 5) such that degraded subjects were more likely to “obey” misleading/false minority feedback and “disobey” majority/true feedback. Thus, they shifted to the other stimulus inappropriately after “true” correct feedback (*t*_4_ = 7.351, *P* = 0.002), kept responding (stayed) on the same stimulus inappropriately after true incorrect feedback (*t*_4_ = −5.656, *P* = 0.005), stayed inappropriately after false correct feedback (*t*_4_ = −5.656, *P* = 0.005), and shifted inappropriately after false incorrect feedback (*t*_4_ = −3.917, *P* = 0.017). See Fig. 4 (G and H).

To better understand these behavioral changes, we used Bayesian hierarchical modeling to fit and compare several computational models to the behavioral data (see Supplementary Methods). The model that best explained the data (see Supplementary Results, table S1, and fig. S5) was one in which predegradation behavior was governed by (i) reinforcement learning (without the need for different learning rates for reward and punishment); (ii) stimulus stickiness, subjects’ tendency to “stick” with their previous choice of stimulus; (iii) side stickiness, subjects’ tendency to stick with their previous choice of side (left or right); and (iv) an additional tendency to make a choice closer to a theoretically optimal action selection, given the subject’s experience to date [optimality being determined by randomized probability matching (RPM); see Supplementary Methods].

**Fig. 5.**
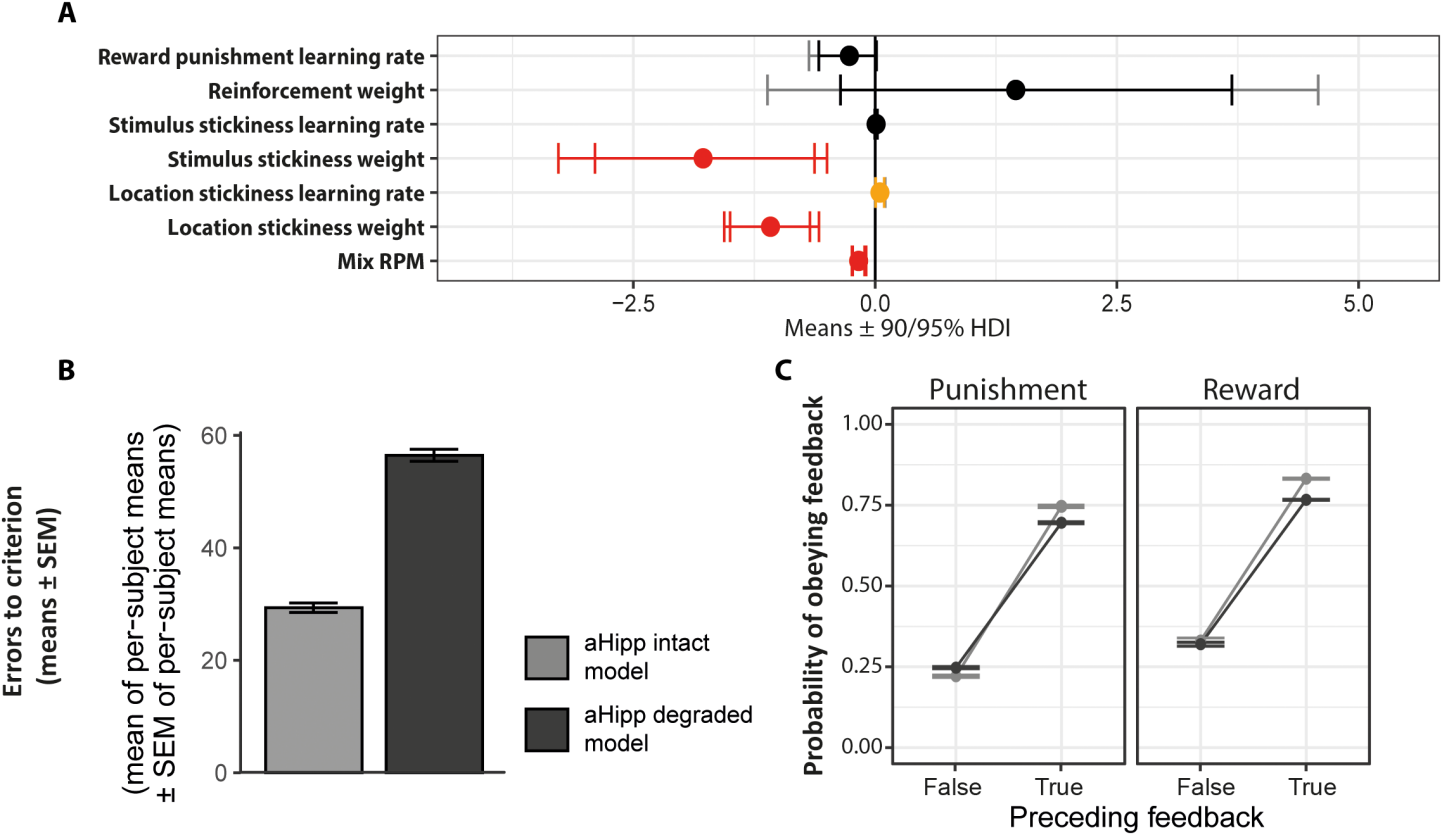
Behavioral impairments caused by aHipp PNN degradation were not attributable to changes in simple reinforcement learning but to changes in perseverative behavior and a reduction in the contribution of higher-order cognitive processes. (**A**) Lesion effects on computational model parameters. For the winning computational model, lesion effects are shown as posterior means (dots), with 95% (outer) and 90% (inner) highest posterior density intervals (HDIs), of the group mean difference (lesion – control) for the parameter of interest (red, 0 ∉ 95% HDI; orange, 0 ∉ 90% HDI; black, 0 ∈ 90% HDI). Conventional behavioral analysis of data simulated using the posterior parameters extracted from the winning computational model (see Supplementary Methods) replicated (**B**) the increased errors to criterion per discrimination (mean of per-subject means ± SEM of per-subject means) and (**C**) the altered feedback responsivity shown by the marmosets (compare Fig. 3, F and G).

Next, we looked to see how aHipp PNN degradation altered the values of these parameters (Fig. 5A). The degradation did not affect reinforcement learning. However, it reduced the degree to which subjects exhibited both stimulus and location stickiness and also reduced their reliance on “optimal” behavior, as judged by the RPM mixing parameter [posterior *P*_zero_ < 0.05 or 0 ∉ 95% highest posterior density interval (HDI; credible interval) for these effects; Fig. 5A]. Simulation of behavior using the computational model replicated the increase in errors to criterion (Fig. 5B) and largely replicated the altered responses to feedback (Fig. 5C) seen after aHipp PNN degradation. Thus, the winning computational model indicated first that aHipp PNN degradation reduces the tendency of marmosets to persist with responding to a chosen side or location, an increase in behavioral switching that is consistent with that seen in schizophrenia (*21*). Second, while intact marmosets used RPM to exhibit behavior that was better (more “optimal”) than what could be explained by just simple reinforcement learning and “stickiness” processes, this ability was reduced by aHipp PNN degradation.

## DISCUSSION

Animal models are unlikely to ever recreate the complex and varied symptom profile of schizophrenia without a better understanding of the cause(s) of the disorder. However, by recapitulating key neurobiological features of schizophrenia, it is possible to determine which neurotransmitter systems and brain circuits can contribute to particular symptoms. This is particularly important for the cognitive symptoms of schizophrenia, which are poorly treated with current therapeutic drugs and where animal model development has been slow to incorporate new clinical findings [see (*22*)]. Here, we used aHipp PNN degradation to recapitulate key neurobiological features of schizophrenia in a primate model, permitting the investigation of neurochemistry and function within human-like prefrontal regions known to be implicated in schizophrenia-related cognitive impairments. Specifically, aHipp PNN degradation caused increased dopamine synthesis in the associative striatum, impaired probabilistic discrimination learning in a task known to depend upon orbitofrontal-striatal circuitry, and increased orbitofrontal dopamine and noradrenaline release.

Increased presynaptic striatal dopamine and hyperactive striatal dopamine transmission has long been understood as fundamental to the development of psychosis. Consequently, similar dopaminergic changes are considered key to animal models of schizophrenia. In rodents, most models consider an increased locomotor response to amphetamine, indicative of striatal hyperdopaminergia akin to the clinical state. Amphetamine-induced dopamine release and the associated locomotor response are mediated primarily by limbic dopamine release in the Acb. Locomotion is induced by intra-Acb administration of amphetamine or dopamine, and dopamine receptor blockade in the Acb inhibits the locomotor response (*23*–*25*). However, repeated PET imaging studies of striatal dopamine perturbations in patients with schizophrenia have demonstrated that the increases in presynaptic dopamine and dopamine synthesis are localized to the associative striatum, not the Acb (*14*, *15*, *26*). This anatomical mismatch has major implications for the ability of animal models to recreate prefrontal dysfunction appropriately and for our understanding of cognitive impairments mediated by the prefrontal cortex, given the heterogeneous connectivity across different prefrontal and striatal subregions.

In this context, the causal findings of the present study in a primate diverge from those reported in a rodent. Degradation of the aHipp PNN caused increases in locomotion and grooming that were exacerbated by an amphetamine challenge but were not associated with increases in extracellular dopamine levels or dopamine synthesis within the Acb. Instead, increased dopamine synthesis was localized to the GP and caudate tail, both parts of the associative striatum. While this is more representative of the clinical state, it raises interesting questions with respect to the increased locomotion/stereotypical grooming observed. In the rodent, locomotion and stereotypies have been localized to the Acb and caudate/putamen, respectively (*23*), while hyperactivity has been observed after caudate body damage in the primate (*27*). It is possible that in the present study, amphetamine modulated dopamine levels within the caudate body; some human studies have identified greater dopamine changes in the dorsal associative striatum (*14*). Nevertheless, while the role of the caudate tail in locomotor activity is not well understood, both the internal GP and external GP have well-defined roles in the regulation of motoric behavior via the “direct” and “indirect” pathways through the basal ganglia, and stimulation of the associative and limbic segments of the primate external GP induces hyperactivity and stereotyped behaviors (such as grooming), respectively (*28*). These behavioral changes may be mediated by dopamine, as GP neurons express dopamine receptors (*29*) and intrapallidal amphetamine injection produces acute stereotypic behavior and hyperactivity in rodents (*30*). In the context of Parkinson’s disease, internal GP dopamine also facilitates movement (*31*); thus, the increased pallidal dopamine synthesis we observed may have been responsible for the increased movement seen in the marmoset locomotion task. Little is known about the role of the GP in schizophrenia, but it has motor, associative, and limbic subdivisions that are connected to corresponding regions in the cortex and striatum (*17*). Importantly, GP volume has been suggested as a diagnostic marker for schizophrenia (*32*), and the GP can regulate dopamine release via direct projections to the substantia nigra pars reticulata, potentially giving it a key role in influencing dopaminergic dysfunction in schizophrenia.

The contribution of dopamine to impaired probabilistic discrimination learning is less clear. While dopamine within OFC-caudate circuitry is implicated in discrimination learning, low levels of OFC dopamine facilitate discrimination and reversal learning (*19*, *33*), while high levels can lead to a perseverative response (*34*, *35*). Thus, the high OFC dopamine seen in the present study as a consequence of aHipp PNN degradation is unlikely to be responsible for the nonperseverative behavioral impairment. On the other hand, increased dopamine synthesis observed in the caudate tail may also have contributed. Neurons in the caudate tail are active during visual discrimination learning and encode stimulus value, and this region can modulate dopamine via projections to the substantia nigra pars reticulata (*36*). Lesions of the caudate tail impair discrimination learning (*37*, *38*), but its role in schizophrenia and the role of dopamine within it are unknown, largely due to poor anatomical localization and identification of this region in human neuroimaging studies [see (*36*)].

Alternatively, the impaired discrimination learning may have been caused by the elevated OFC noradrenaline. Increased OFC noradrenaline is proposed to increase cognitive flexibility by facilitating behavioral exploration in situations of contingency change (*39*). In deterministic tasks, this would be expected to reduce inappropriate perseveration and be advantageous for performance, and increased OFC noradrenaline does improve deterministic reversal learning (*40*). However, in tasks in which contingent relationships are probabilistic and, therefore, error feedback can be misleading, as in the present study, an increase in responding according to the probabilistic feedback would cause decreased perseveration and increased lose-shift and decreased win-stay behaviors, as we observed, and slow adaptive learning overall, as is seen in patients with schizophrenia (*20*, *41*). Related to this, orbitofrontal noradrenaline is also associated with the updating of goal-directed behaviors (*42*). Instrumental learning can be driven by both goal-directed processes (where the action is associated with a representation of the valued outcome) and/or a gradual process of reinforcement (e.g., habit learning) without explicit representations of the outcome. Our computational modeling indicated that simple reinforcement learning was being used here but that a deficit in it was not responsible for the impaired discrimination learning, as parameters governing this process were unaltered by aHipp PNN degradation. Instead, in addition to reductions in repetition behaviors (perseveration or stickiness), the behavioral changes were also driven by reductions in the contribution of a higher-order cognitive process (modeled here by a theoretically optimal RPM method), which may therefore represent deficits in the goal-directed action system. Deficits in goal-directed behavior are fundamental to the altered cognition observed in schizophrenia (*43*), and the pattern of goal-directed impairments alongside intact reinforcement learning replicates that seen in the disorder (*44*). While the role of noradrenaline in these processes requires further investigation, noradrenaline has a widespread distribution across the brain, and it is noteworthy that K^+^-evoked noradrenaline release in the Acb was also elevated, albeit nonsignificantly, given the elevated noradrenaline levels found in the cerebrospinal fluid and serum of patients with schizophrenia (*45*, *46*), and the positive association of noradrenaline with stress—a known trigger for schizophrenia (*47*). Last, it is possible that the mechanistic basis via which hippocampal PNN destruction caused these behavioral/cognitive changes is independent from the alterations in neuromodulators such as dopamine and noradrenaline that this PNN degradation demonstrably caused. Thus, we cannot rule out the role of altered information flow via direct hippocampal-OFC projections (*48*) or the contribution of local hippocampal circuit dysfunction. However, there is little evidence that the hippocampus is required for visual object discrimination learning, only the retention of a learnt discrimination—the opposite pattern of impairment seen here (*49*).

Together, these data demonstrate causally that a loss of aHipp PNNs is sufficient to induce an overactive dopamine system in the associative striatum, which is purported to underlie the positive symptoms of schizophrenia. The same loss of aHipp PNNs also causes dysfunction in prefrontal systems considered critical for some of the cognitive symptoms of schizophrenia, implicating dopamine and noradrenaline increases in the OFC as potential causes of these symptoms. However, the failure of the current D2 antagonist treatments to ameliorate impaired cognition highlights the potential importance of noradrenaline and suggests that inhibiting the effects of OFC noradrenaline may provide a potential therapeutic avenue. This approach highlights the importance of primate translational neuroscience for the development of a mechanistic understanding of prefrontal cortex function and of alterations seen in schizophrenia, with a view to developing new treatments for disorders of major personal and societal consequence.

## MATERIALS AND METHODS

### Study design

This manuscript uses three cohorts of marmosets to examine how degradation of the primate hippocampal PNN alters striatal and prefrontal functions that are important in schizophrenia symptomatology. The study to address the neurochemical alterations in the Acb and OFC used five marmosets, and a further five performed the discrimination learning task, of which four also completed the amphetamine-induced activity paradigm. Last, three animals completed the neuroimaging study and an additional four were used to quantify the degradation histologically (alongside two from the microdialysis cohort). All behavioral measures were analyzed by a computer or an individual blind to any drug treatments, and no data were excluded. Animals were assigned randomly to their experimental cohorts and acted as their own behavioral and neurochemical controls. Group sizes were selected according to power analyses from previous studies.

### Marmosets

Seventeen marmosets were bred on site at the University of Cambridge Marmoset Breeding Colony and housed in male/female pairs (10 females and 7 males; males were vasectomized). They were kept in a 12-hour light-dark cycle (lights on at 7:00 a.m., lights off at 7:00 p.m.) in a controlled environment of 22 ± 1°C and 50 ± 1% humidity. Their cages contained a variety of environmental enrichment aids including suspended ladders, ropes, and wooden branches to climb and swing on, and boxes to play in that were varied. Eight marmosets (five females and three males) took part in the discrimination learning experiment and other tasks not reported here and were fed 20 g of MP.E1 primate diet (Special Diet Services) and two pieces of carrot on 5 days per week after the daily behavioral testing session, with simultaneous free access to water for 2 hours. On weekends, their diet was supplemented with fruit, rusk, malt loaf, eggs, bread, live insects, and treats, and they had free access to water. All other animals received a rotation of the foods above each day. All procedures were performed in accordance with the UK Animals (Scientific Procedures) Act, 1986, and the University of Cambridge Animal Welfare and Ethical Review Board. See Table 1.

**Table 1.** Summary of monkeys that took part in these studies. An X indicates that the subject took part in that element of the study. Blank spaces indicate that an animal did not participate in a given element. Subjects 14 to 17 had unilateral degradations and took in part in non-OFC prefrontal microdialysis not reported here. An asterisk (*) indicates animals that contributed to the histological quantification in Fig. 1C (see histological methods). The number in brackets indicates how many times each animal received degradation 4 to 5 weeks apart. All unilateral aHipp degradations were in the left hemisphere, with the right aHipp receiving vehicle simultaneously.

Marmoset	Degradation	OFC dialysis	Acb dialysis	Discrimination learning	Amphetamine-induced locomotion	[^18^F]-DOPA PET
1 (female)	Unilateral (2)	X	X			
2* (male)	Unilateral (2)	X	X			
3* (female)	Unilateral (2)	X	X			
4 (female)	Unilateral (2)	X	X			
5 (male)	Unilateral (1)	X				
6 (female)	Bilateral (3)			X	X	
7 (male)	Bilateral (2)			X	X	
8 (female)	Bilateral (3)			X	X	
9 (male)	Bilateral (3)			X	X	
10 (female)	Bilateral (4)			X		
11 (male)	Bilateral (4)					X
12 (female)	Bilateral (4)					X
13 (female)	Bilateral (4)					X
14* (male)	Unilateral (2)					
15* (male)	Unilateral (2)					
16* (female)	Unilateral (1)					
17* (female)	Unilateral (2)					

### Cannulation surgery

Subjects were premedicated with Alfaxan (alfaxalone, Zoetis UK, 0.15 to 2 ml of a 10 mg/ml solution, intramuscularly) and given a long-lasting prophylactic analgesic [Metacam; 0.075 ml of meloxicam (50 mg/ml), subcutaneously; Pfizer]. They were intubated and maintained on isoflurane gas anesthetic (flow rate, 2.0 to 2.5% isoflurane in 0.3 liters/min O_2_; Novartis Animal Health UK) and placed in a stereotaxic frame modified for the marmoset (David Kopf). Animals were constantly monitored clinically and with pulse oximetry/capnography. Cannulae (Plastics One, Roanoke, VA) were implanted into the aHipp [double 15-mm-long cannula, 1.4 mm apart; anteroposterior (AP) +6; lateral-medial (LM) ±5.35/6.75; ventral (V) +5] with coordinates adjusted in situ according to cortical depth where necessary (*50*).

Postoperatively, and when fully recovered, all monkeys were returned to their home cage and then received the analgesic meloxicam (0.1 ml of a 1.5 mg/ml oral suspension; Boehringer Ingelheim) for 3 days as well as 10 days of “weekend diet” and water ad libitum to allow complete recovery before returning to testing. Cannulae were cleaned at least once a week (and caps and cannula blockers changed) to ensure that the cannula site remained free from infection.

### Infusions

Every 4 to 5 weeks, animals received infusions of Chondroitinase ABC from *P. vulgaris* (Sigma-Aldrich, UK) or vehicle (BSA, Sigma-Aldrich, UK) into the aHipp via their hippocampal cannulae. This is because rodent studies indicate that 6 weeks after enzyme injections, the PNNs start to regrow; therefore, redoing the infusion at 4 to 5 weeks prevented this regrowth from occurring (*51*). The use of chronically implanted cannulae allowed animals to act as their own control and reduced experimental variation caused by individual differences. All sterile infusions were conducted with the monkey held gently in a familiar researcher’s hand. The caps and cannula blockers were removed from the guide, and the site was cleaned with either 70% alcohol or Hibiscrub (Regent Medical, UK). A sterile injector (Plastics One), connected to a 2-μl gas-tight syringe in a syringe pump, was inserted into the guide cannula for the duration of the infusion. Following the infusion, the injector was left in place for a further minute to allow the drug to diffuse before injector removal. Sterile cannula blockers and caps were replaced, and the marmoset was returned to its home cage. Vehicle infusions consisted of 0.1% BSA in saline infused at the rate of 0.3 μl/min for 2 min in the lateral aHipp site and 2 min and 20 s in the medial aHipp site. Chondroitinase ABC infusions consisted of 0.05 U/μl in 0.1% BSA and were otherwise identical.

### [^18^F]-DOPA PET

To determine the effects of aHipp PNN degradation on striatal dopamine synthesis, we used PET imaging with the radioligand [^18^F]-DOPA. As it has been demonstrated that baseline striatal dopamine synthesis (*52*) varies between individuals, the monkeys were scanned both before and after degradation so that each monkey could act as its own control when assessing the effects of aHipp PNN degradation on striatal dopamine synthesis. PET scans always occurred 2 weeks after the first infusion of either vehicle or chondroitinase [and 1 week after magnetic resonance imaging (MRI) scanning]. One hour before the [^18^F]-DOPA injection, each animal received carbidopa (10 mg/kg; Biotechne, UK) in 10% glucose (per os; Sigma-Aldrich, UK) to reduce peripheral decarboxylation of [^18^F]-DOPA, thereby preferentially increasing the bioavailability of [^18^F]-DOPA relative to radiolabeled metabolites, which can confound measurements by crossing the blood-brain barrier (*53*). Animals were then premedicated as before. The tail vein was cannulated (Intraflon 2 intravenous catheter attached to a Lock Stopper with an injectable membrane; Vygon) and flushed with 0.5 ml of heparinized saline, and the monkey was subsequently intubated and maintained on isoflurane gas anesthetic (flow rate: 2.0 to 2.5% isoflurane in 0.3 liters/min O_2_; Novartis). Animals were imaged using a micro PET Focus 220 scanner (Concorde Microsystems, Knoxville, TN). The brain was located centrally in the field of view of the scanner to maximize sensitivity and spatial resolution, and [^18^F]-DOPA (scan 1, 41.6 ± 5.8 millibecquerels; scan 2, 41.3 ± 8.1 millibecquerels) was injected into the tail vein as a bolus over 10 s, followed by 0.3 ml of heparinized saline flush. List-mode PET data acquisition for 90 min started at the same time as the injection of [^18^F]-DOPA. List-mode PET data were histogrammed into 42 sinograms and then reconstructed using Fourier rebinning (*54*), followed by two-dimensional ordered subset expectation maximization (*55*) with 6 iterations and 16 subsets. As transmission scanning was not feasible, attenuation correction used a mean non–attenuation-corrected [^18^F]-DOPA image to determine a body outline within which a uniform attenuation coefficient (0.096 cm^−1^) was ascribed, and this was combined with a standard attenuation map of the carbon fiber bed. Corrections were also applied for randoms, dead time, normalization, sensitivity, and decay. Postsmoothing with a 2-mm full width at half maximum Gaussian was applied to the images. A mean [^18^F]-DOPA image of each scan was manually, rigidly registered to an anatomical magnetic resonance (MR) image of the same animal, and thence, the dynamic [^18^F]-DOPA image series was registered to the MR image. A standard set of regions of interest (ROIs) was defined on this MR image via nonrigid registration of an ROI atlas delineated on a template image described previously (*56*). For each ROI, reference tissue Patlak analysis (*57*) with an occipital ROI as the reference region (*58*) was applied in the time period of 35 to 65 min after injection, as preliminary analysis for a whole-striatum ROI indicated that this period provided the best approximation to a linear fit for determination of *k*_3_^s^, an estimate of the rate at which [^18^F]-DOPA is decarboxylated to [^18^F]-fluorodopamine. For illustrative purposes, −log_10_(*P* value) maps were made to depict the statistical significance of differences in regional *k*_3_^s^ between baseline and postdegradation scans across animals, with bilateral ROI data pooled and *P* values determined using a paired *t* test. The sign of the mean difference was used to produce separate maps for *k*_3_^s^ increases and decreases.

Synthesis of [^18^F]-DOPA from an aqueous solution of [^18^F] followed the cassette-based procedure (ABX, Germany) on a FASTlab synthesizer (GE Healthcare, UK). The identification and purity of [^18^F]-DOPA were determined using an X-Terra Waters column (4.6 by 250 mm, 5 mm) eluting with aqueous trifluoroacetate (1.22 g/liter; phase A) in acetonitrile (phase B) at a flow rate of 1 ml/min using a gradient as follows: 0 to 10 min, 98% A; 10 to 13 min, 98 to 95% A; 13 to 20 min, 95% A; 20 to 35 min, 95 to 5% A; 35 to 37 min, 5% A. The radiochemical purity of the [^18^F]-DOPA produced is normally greater than 99%.

### Magnetic resonance imaging

High-resolution anatomical MR images were obtained with a 9.4-T Bruker BioSpec 94/20 system (Bruker, Coventry, UK). Animals were anesthetized with isoflurane (1% in 0.3 liters/min O_2_). The sequence used was a magnetization transfer prepared multigradient echo sequence (repetition time/echo time/flip angle, 25/2.4 to 13.4 ms/6°) with an image matrix of 256 by 160 by 128 for a 175-μm isotropic resolution in 7 min and 28 s. An eight-channel phased array coil designed for marmosets (RAPID Biomedical, Germany) was used for signal reception, and the standard system body coil was used for signal transmission.

Images were processed using SPM12 (Wellcome Trust Center for Neuroimaging, UCL, UK) with the SPMMouse toolbox for animal data (*59*). The multigradient echo images were combined with a signal-weighted average over echoes and aligned with tissue probability maps derived from marmosets from the same colony (*60*) and segmented into gray matter, white matter, and cerebrospinal fluid. DARTEL (*61*) was used for image registration and to create population templates. MRI scans always occurred 1 week after the first infusion of either vehicle or chondroitinase.

### In vivo microdialysis

Two weeks after unilateral aHipp PNN degradation (and 1 week after MRI), four animals were anesthetized with isoflurane and acutely implanted with commercially available BASi brain microdialysis probes (BASI MD-2200, BR-2, Bioanalytical Systems) into the OFC targeting area 11 (AP +17 mm; LM ±3 mm; 1.2 mm from the base). Harvard microsyringe pumps with 2.5-ml gas-tight syringes were used to perfuse artificial cerebrospinal fluid through the dialysis probe at a flow rate of 1.0 μl/min. The artificial cerebrospinal fluid had the following composition: 147 mM NaCl, 3.0 mM KCl, 1.3 mM CaCl_2_, 1.0 mM MgCl_2_, 0.2 mM NaH_2_PO_4_, and 1.3 mM Na_2_HPO_4_. After allowing 3 hours and 50 min for the implanted probes to equilibrate, dialysate fractions were collected every 25 min into 2 μl of 0.01 M perchloric acid. After a baseline sample, monkeys received a 75 mM K+ challenge for 10 min, which was followed by a further three baseline samples. Samples were stored at −80°C before being analyzed using reversed phase high-performance liquid chromatography and electrochemical detection by Sygnature Discovery UK (www.sygnaturediscovery.com/). Recovery is described above. Degradation was repeated 4 weeks after the first infusion, and the timeline was repeated. Thus, animals were MRI scanned 1 week after degradation and microdialysis was repeated a week later (2 weeks after aHipp PNN degradation), this time targeting the Acb [AP +12.5 mm; lateral (L) ±2.3 mm; dorsoventral (DV) +9 mm]. Both procedures were conducted under isoflurane anesthesia. After the second procedure was complete, the anesthesia was switched to pentobarbital for euthanasia.

### Discrimination learning

Subjects (*n* = 5) completed a probabilistic discrimination learning task, as described previously (*19*), which consisted of multiple discriminations, each comprising two abstract multicolored stimuli presented on a touch-sensitive computer screen. All monkeys were trained to enter a clear Perspex transport box for marshmallow reward, habituated to the testing apparatus, and trained to respond to the touchscreen, as described previously (*62*). The correct stimulus was usually associated with a 5-s banana milkshake reward and occasionally punished with a 0.3-s, 100-dB loud noise, and vice versa for the incorrect stimulus, according to the probability ratio of the discrimination (90%:10%, 80%:20%, and 70%:30%). Each session (one per day) consisted of 30 trials, and the discrimination was considered learned when they attained a criterion of 90% or more correct choices in one session. A new discrimination was then started the next day. Subjects’ ability to learn was assessed behaviorally by how many incorrect choices they made in total while learning a discrimination and by their win-stay/lose-shift performance. (When animals “win,” i.e., attain reward, one potentially appropriate behavior is to “stay” with the rewarded stimulus and choose it again on the next trial. Conversely, when animals “lose,” i.e., receive punishment, a potentially appropriate behavior is to “shift” to the other stimulus. However, this behavior becomes less appropriate with probabilistic feedback.)

### Amphetamine-induced activity and scoring

**Fig. 6.**
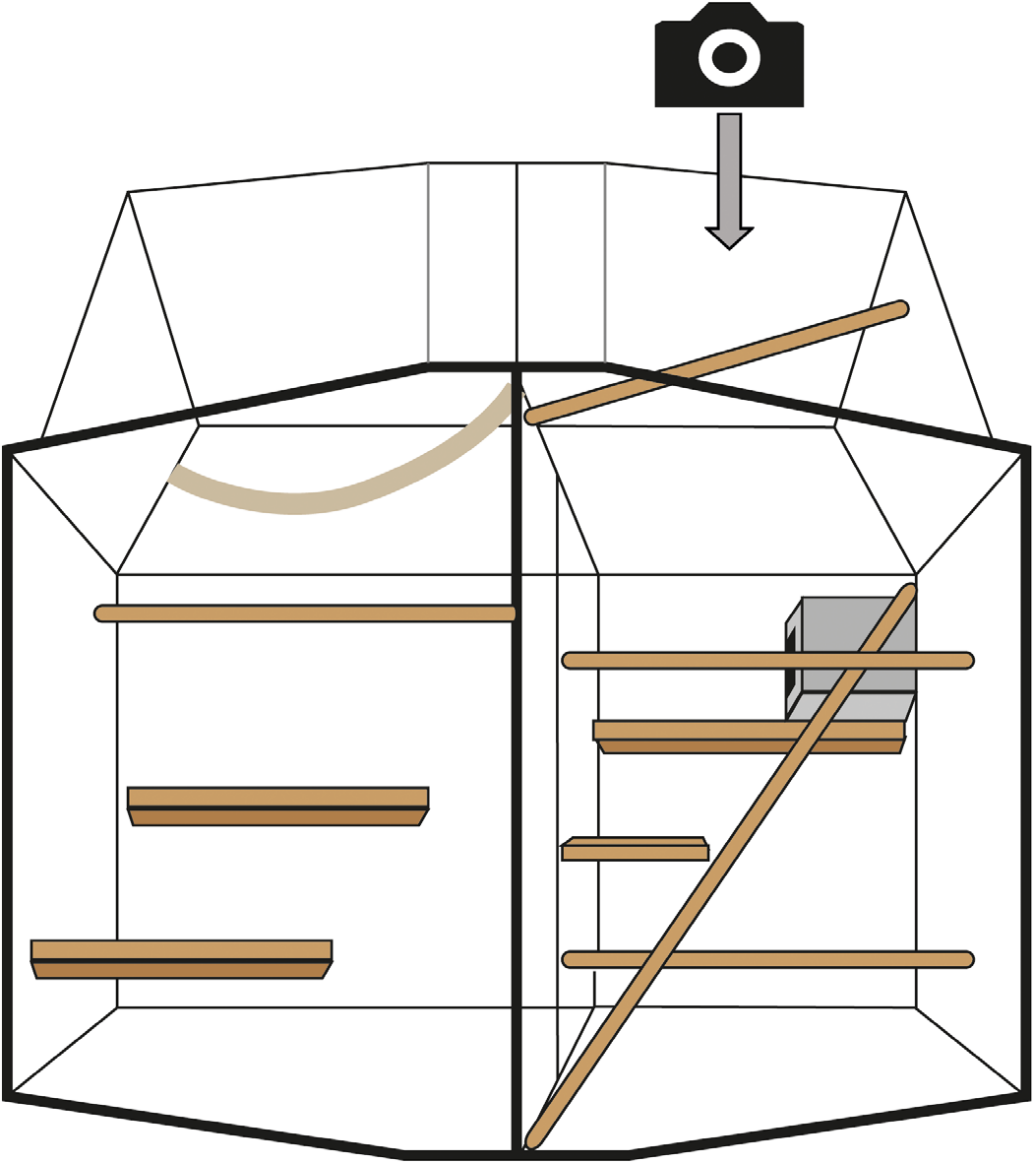
Amphetamine locomotion and stereotypy assessment. Animals were segregated into the top half of their home cage (depicted) and a video camera placed on top of the transparent roof pointing down. Behavior was recorded for 45 min before a challenge (saline or amphetamine) and for 2.5 hours afterward.

Animals were segregated into the top half of their home cage with their partner, and a video camera was positioned on top looking downward (see Fig. 6). They were recorded for 45 min before receiving an injection of *d*-amphetamine (0.5 mg/kg; Sigma-Aldrich, UK) or saline intramuscularly. They were then recorded for a further 2.5 hours. Their activity for the entire video duration was then scored. Every 5 min, their behavior was scored for 1 min with Jwatcher (www.jwatcher.ucla.edu/). The behaviors scored were the amount of time they spent moving around the cage (“locomotion”; milliseconds) and the time spent compulsively self-grooming (“grooming”; milliseconds). If they were not visible (for example, due to being under a shelf) during a 1-min slot, the nearest minute where they were visible was scored instead. Data for each animal were initially normalized to the combined values for that animal, across all degradations and drugs, using *z*-score normalization. Following normalization, the data for each period for each animal were summed from 5-min bins into 15-min bins and then smoothed using the “lowess” smoothing function in MATLAB. A mean was then calculated for each of these periods. For analyses where time was not a factor, the data were summed over all time bins and no smoothing was used.

### Postmortem histology and degradation assessment

All monkeys were premedicated with Alfaxan (alfaxalone, Zoetis UK, 0.15 to 2 ml of a 10 mg/ml solution, intramuscularly) and humanely euthanized with Euthatal (1 ml of a 200 mg/ml solution, pentobarbital sodium, intravenously; Merial Animal Health Ltd.) before being perfused transcardially with 500 ml of ice-cold 0.1 M phosphate-buffered saline (Sigma-Aldrich), followed by 500 ml of 4% formaldehyde solution (VWR International) over ∼15 min. The entire brain was then removed and placed in 0.01 M phosphate-buffered saline-azide overnight before being transferred to a 30% sucrose solution for at least 48 hours.

For verification of cannulae and microdialysis probe placement, 60-μm coronal sections of the brain were cut using a freezing microtome, the cell bodies were stained using cresyl violet (FD Cresyl Violet, no. PS102-01, FD Neurotechnologies), and the sections were imaged using standard bright-field microscopy. For each animal, cannula locations were schematized onto drawings of standard marmoset brain coronal sections, and composite diagrams were then made to illustrate the extent of overlap between animals.

For degradation assessment, two staining approaches were used to stain parvalbumin (PV)–positive interneurons and the chondroitin component of the PNN that is degraded by the chondroitinase. Initially, fluorescence staining was performed. The PV was stained using an anti-PV rabbit primary antibody (ab11427; Abcam, UK; 1:1000), and the PNNs were stained using primary biotinylated lectin from *Wisteria floribunda* (WFA; L1516, 2 mg, Sigma-Aldrich, UK; 20 μl/ml) by incubating them together overnight at room temperature. Corresponding fluorescent secondary antibodies, Alexa Fluor 488 goat anti-rabbit (ab150077; Abcam, UK; 1:2000) and Texas red streptavidin (SA-5006-1; VectorLabs, US; 10 μl/ml), were applied with a 2-hour incubation at room temperature before coverslipping with Vectashield DAPI for imaging. We then switched to 3,3′-diaminobenzidine (DAB) staining for long-term imaging.

For DAB staining, PNNs were stained using biotinylated lectin from *W. floribunda* incubated for 24 hours at 4°C, followed by Vectastain Elite ABC Kit (Peroxidase PK-6100, VectorLabs, US) for 1 hour at room temperature, and lastly reacted with SIGMAFAST DAB (brown; D4393-50SET; Sigma-Aldrich, UK) for 1 min. To costain the PV interneurons, slices were incubated overnight at room temperature with an anti-PV rabbit primary antibody, then a goat-anti-rabbit secondary antibody (ab6720) for 2 hours at room temperature, and ExtrAvidin-Perioxidase as a tertiary antibody (E2886; Sigma-Aldrich, UK) for 1.5 hours at room temperature before reacting with ImmPACT SG (gray; SK-4705, VectorLabs) for 45 s. Sections were then mounted, dehydrated, and coverslipped with DPX (06522; Sigma-Aldrich) before imaging using a Hamamatsu Nanozoomer.

To quantify the effectiveness of the chondroitinase enzyme at degrading PNNs in the marmoset, further analysis was conducted in MATLAB using the Image Processing Toolbox to identify and count PNNS from the brown DAB–stained pixels. Nanozoomed images were cropped to the hippocampus, the caudate tail, and the GP, and the range of RGB values that was appropriate to the stained PNN within each image was selected (using Impixel). This accounted for any variation in staining across sections. Each image underwent color thresholding to extract only pixels within this range of values, and a two-dimensional moving average (smoothing; 15 pixel by 15 pixel) was taken over the image to remove any outlying pixels before the number of pixels was quantified as a measure of PNN integrity and, therefore, degradation.

This was done for each area on the left (degraded) and right (intact) sides of six unilaterally degraded marmosets, and a ratio of the average across left and right sides was calculated before analysis with a paired *t* test. Hippocampal sections were selected according to those that appeared most visually degraded but had minimal tissue damage from the cannulae. To prevent bias caused by potential staining artifacts or experimenter bias, the sections immediately anterior and posterior to the chosen section were also selected and an average was created over all three. The caudate was analyzed using the same sections as the hippocampal analysis, but the GP was too anterior. Instead, the section with the largest component of the GP was selected and the anterior/posterior sections were used for averaging as before.

### Data and statistical analysis

Behavioral, neurochemical, and histological statistical analyses were performed with IBM SPSS statistics (version 27, IBM, US) and MATLAB 2024a, with figures produced within Microsoft Excel and Adobe Illustrator. Computational modeling and graphical representation used Stan, RStan, and R version 2.21.2. For all ANOVAs, *F*, *P*, and *t* values are reported throughout. Statistical significance is indicated by *P < 0.05, **P < 0.01, and ***P < 0.001 in all figures.

## Supplementary Materials

### This PDF file includes:

Supplementary Methods Tables S1 to S5 Figs. S1 to S7 References
