# Supplementary material for: Hippocampal perineuronal net degradation identifies prefrontal and striatal circuits involved in schizophrenia-like changes in marmosets
Source: Sci Adv. 2025 Apr 18;11(16):eadu0975. doi: 10.1126/sciadv.adu0975 (PMC12007587; doi:10.1126/sciadv.adu0975)
Supplement: Supplementary file 1 — Supplementary Methods Tables S1 to S5 Figs. S1 to S7 References [file sciadv.adu0975_sm.pdf]

Science Advances
AAAS

Supplementary Materials for

Hippocampal perineuronal net degradation identifies prefrontal and striatal circuits involved in schizophrenia-like changes in marmosets

Miriam A. Gwilt *et al.*

Corresponding author: Hannah F. Clarke, hfc23@cam.ac.uk

Sci. Adv. **11**, eadu0975 (2025)
DOI: 10.1126/sciadv.adu0975

This PDF file includes:

Supplementary Methods
Tables S1 to S5
Figs. S1 to S7
References

S1. Supplementary methods: Computational modelling

S2. Overview of hierarchical modelling approach

We fitted a family of computational models to the behavioural data, using Bayesian hierarchical modelling, verifying convergence. We used bridge sampling to compare the performance of different models (automatically penalizing for model complexity), and calculated the area under the receiver operating curve (AUROC) for each model to provide a real-world measure of goodness of fit. For the winning model, we extracted posterior parameter distributions of interest, as well as verifying parameter recovery and generating simulated behaviour from that computational model (using the posterior mean parameters).

We embedded the experimental within-subjects design within the hierarchical models. For every model parameter separately, a subject's individual parameter value was drawn from a distribution having a mean specific to its group/condition (i.e. for each parameter, estimating separate means before and after the lesion), and a subject-specific component representing intersubject variation (whose variance was also estimated).

S3. Models tested

S4. Fit to behaviour

For all models, we calculated a trial-by-trial probability of the response being on the left-hand side, and fitted that to the binary behavioural data via the Bernoulli distribution (or a log-odds equivalent).

S5. Action probability calculation

For most models, we calculated the probability of each of the two actions from an "action tendency" $\boldsymbol{a}$ via the softmax function:

$$p_i = \frac{e^{a_i}}{\sum_j \ e^{a_j}} = \text{softmax}_i(a)$$

For some we used the mathematical equivalence for the special case of this two-choice situation, via log odds (O) and fitting via the Bernoulli logit distribution. This is faster but exactly equivalent. Expressed for actions L(eft) and (R)ight, this is:

$$\ln O_L = \ln \frac{p_L}{1 - p_L} = \ln \frac{p_L}{p_R} = \ln[\frac{e^{a_L}}{e^{a_L} + e^{a_R}} \frac{e^{a_L} + e^{a_R}}{e^{a_R}}] = \ln \frac{e^{a_L}}{e^{a_R}} = \ln e^{a_L} - \ln e^{a_R} = a_L - a_R$$

The action tendency comprised components representing different psychological processes, each typically weighted by a process-specific softmax inverse temperature β:

$$a = \beta_1 x_1 + \beta_2 x_2 + ..$$

For example, a model combining reinforcement, stimulus stickiness, and location stickiness (all described below) would use:

$$a = \beta_{reinf} v + \beta_{stim} s + \beta_{loc} l$$

For a few models, action probabilities were calculated slightly differently (see Bayesian updating and randomized probability matching [RPM] models below).

S6. Reinforcement learning (RL)

This component embodies traditional learning from reinforcement. Models maintained stimulus "values" (action values, Q values) (*63*, *64*), initialised at 0.5 (*19*). When a stimulus was chosen, its value was updated according to ensuing reinforcement $\lambda \in \{0, 1\}$, via a learning rate α_{reinf} constrained to the range [0, 1], and a simple delta rule (*65*):

$$\Delta v = \alpha_{\text{reinf}}(\lambda - v)$$

or, equivalently:

$$v_{\text{new}} \leftarrow (1 - \alpha_{\text{reinf}})v_{\text{old}} + \alpha_{\text{reinf}}\lambda$$

This process had an associated softmax inverse temperature, β_{reinf}, constrained $[0, \infty)$. Some previous studies have found the constraint $\beta_{\text{reinf}} = 1$ to be parsimonious (*19*, *66*). In the present study, we used β_{reinf} as a free parameter throughout (*67*) (in which study, expressed as τ), because as values v are constrained to be [0, 1], $\beta = 1$ would constrain the maximum preference probabilities resulting from this process alone to approximately [0.269, 0.731], i.e. prohibit very sharp reinforcement-based preferences.

In other variations, we used separate learning rates for reward (α_{rew}) and punishment (α_{pun}), chosen according to the outcome of a given trial.

S7. Global reward trace

In this modified RL model, with additional parameters α_{R} and w_{R}, prediction error is modified by the longer-term history of reward (*68*). The global reward trace g began at 0, and was updated after reinforcement according to:

$$\Delta g = \alpha_{\text{R}}(\lambda - g)$$

Prediction error, normally $\lambda - v$, was modified:

$$\Delta v = \alpha_{\text{reinf}}(\lambda + w_{\text{R}} \cdot g - v)$$

Constraints were [0, 1] for α_{R}, and [–1, 1] for w_{R} (*68*). In previous work, w_{R} was empirically positive, indicating that a recent history of reward enhances learning from reward (*68*). This procedure removes the normal [0, 1] constraint on v.

S8. *Stimulus "stickiness"*

This component embodies a tendency to choose a stimulus simply because it has been chosen recently. In "full" stimulus-stickiness models (*19*, *66*), with parameters α_{stim} and β_{stim}, stimulus "stickiness" values s (associated with each stimulus) began at 0 and updated after choice according to:

$$\Delta s_i = \alpha_{\text{stim}}(S_i - s_i)$$

where S_i is 1 if stimulus i was chosen on that trial, and 0 otherwise. The parameter β_{stim} is a softmax inverse temperature, as above. Constraints were [0, 1] for α_{stim}, and $[0, \infty)$ for β_{stim}; previous work has allowed for the theoretical possibility of "antistickiness" ($\beta_{\text{stim}} < 0$) (*67*) but there is strong evidence for positive values in the present species and task setting (*66*). Stimulus stickiness was not reset across sessions, although was intrinsically reset when a new discrimination began.

In "one-back" stimulus-stickiness models (*67*), with parameter β_{stim}, only the most recent trial was considered, i.e. $s_i = S_i$, equivalent to $\alpha_{\text{stim}} = 1$.

S9. *Location (side) "stickiness"*

This component embodies a tendency to choose a location simply because it has been chosen recently. In "full" location-stickiness models (*19*, *66*), with parameters α_{loc} and β_{loc}, location "stickiness" values l (associated with each side/location) began at 0 and updated after choice according to:

$$\Delta l_i = \alpha_{\text{loc}}(L_i - l_i)$$

where L_i is 1 if location i was chosen on that trial, and 0 otherwise. The parameter β_{loc} is a softmax inverse temperature, as above. Constraints were [0, 1] for α_{loc}, and $[0, \infty)$ for β_{loc}; again, there is strong prior evidence for $\beta_{\text{loc}} > 0$ in the presently relevant context (*66*). Location stickiness was not reset across sessions.

In "one-back" location-stickiness models (*67*), with parameter β_{loc}, only the most recent trial was considered, i.e. $l_i = L_i$, equivalent to $\alpha_{\text{loc}} = 1$.

S10. *Side bias*

In models with a fixed side bias b, constrained [–1, 1], b represented bias towards the right-hand side (0 = no bias). The bias entered as a component of the final action tendency without a further inverse temperature parameter (*68*). A "side bias only" model served as an extremely simple (near-null-hypothesis) control model.

S11. *Lapse to random choice*

In some variations, we applied a lapse parameter ξ, constrained [0, 1], being the probability that choice lapsed to random selection, e.g. after the `bandit4arm_lapse()` model of hBayesDM (*69*):

$$p_{i,\text{new}} = \xi \frac{1}{n} + (1 - \xi)p_{i,\text{old}}$$

where n is the number of actions available.

S12. *Randomized probability matching (RPM) models*

Randomized probability matching (RPM) was developed by (*70*) to discover and exploit the optimal response. It is intrinsically Bayesian and involves counting successes and failures for each option on offer. The model is described in detail in (*19*). RPM is intrinsically parameter-free; we used a parameterized version. We ran one model using RPM as the sole decision process, modified via a softmax inverse temperature parameter β_{RPM} (*19*).

Other models blended RPM with other (e.g. RL or combined) processes, via a mixture parameter m constrained [0, 1] that combined probabilities from the two processes directly:

$$p_{\text{action}} = m \cdot p_{\text{action|RPM}} + (1 - m) \cdot p_{\text{action|other}}$$

We blended RPM in this fashion with a simple RL model (α_{reinf}, β_{reinf} alone) and with leading candidate models involving RL, stimulus stickiness, and side stickiness.

In other models, instead of a mixing parameter, we fed RPM-based action tendences (as log odds) into the same softmax process as before, with an inverse temperature β_{RPM} for this component. (For the two-choice case, softmax is "neutral" with respect to its inputs if the inputs are given as natural log odds and the inverse temperature is 0.5; the outputs are then probabilities corresponding to the log-odds inputs. We used natural log odds clipped to [−20, +20] to avoid infinite-value overflow.)

S13. Simple Bayesian update model

This standalone model was less sophisticated than RPM. It involves a prior belief about reinforcement likelihood and stimuli occurring in pairs (this being potentially less plausible), and a response allocation strategy that is less sophisticated than RPM. In this model, the single free parameter was p(reward | correct), denoted p_{rc}. The model assumed symmetry such that p(reward | incorrect) = $p_{\text{ri}} = 1 - p_{\text{rc}}$, as was the case experimentally. Derived probabilities were p(nonreward | correct) = $p_{\text{nc}} = 1 - p_{\text{rc}}$, and p(nonreward | incorrect) = $p_{\text{ni}} = 1 - p_{\text{ri}}$. Simulated subjects initially considered stimuli in a novel pair to be equiprobable (p = 0.5 each) and subsequently updated the (natural) log odds of the selected stimulus being correct (o_s) after each trial according to Bayes' rule:

$$o_s \leftarrow \begin{cases} o_s + \log(p_{\text{rc}}) - \log(p_{\text{ri}}) \text{if rewarded} \\ o_s + \log(p_{\text{nc}}) - \log(p_{\text{ni}}) \text{if not rewarded} \end{cases}$$

following

$$\log(\text{posterior odds}) = \log(\text{prior odds}) + \log(\text{likelihood ratio})$$

$$\log(\text{likelihood ratio}) = \log[P(\text{D}|\text{H})] - \log[P(\text{D}|\neg\text{H})]$$

Simulated subjects updated the log odds of the unchosen stimulus being correct (o_u) in complementary fashion, according to log_odds(¬A) = –log_odds(A):

$$o_u = -o_s$$

They then chose in direct proportion to the calculated probability of each stimulus being correct on a given trial.

S14. *Experience-weighted attraction (EWA) model*

As a standalone model, we implemented a standard experience-weighted attraction model (*71*, *72*), as in (*67*). It has three parameters: φ (phi), ρ (rho), and β (beta). The "experience weight" of a choice or stimulus that is chosen, $n_{c,t}$, is updated according to the experience decay factor ρ (range $0 < \rho < 1$) [*(11)* equation 1, after *(10)* equation 2.1]:

$$n_{c,t} \leftarrow n_{c,t-1}\,\rho + 1$$

The experience weight is a measure of how often the subject has experienced the action (e.g. chosen a stimulus)—the "number of 'observation-equivalents' of past experience" (*71*). It can increase unbounded.

The value v of a choice is updated according to the outcome λ, and the decay factor for previous payoffs φ (range $0 < \varphi < 1$) [(*72*) equation 2; compare (*71*), equation 2.2, who use capital Φ in place of lower-case φ, and have a more complex payoff term involving π]:

$$v_{c,t} \leftarrow (v_{c,t-1}\,\varphi\, n_{c,t-1} + \lambda_{t-1}) \,/\, n_{c,t}$$

The payoff decay factor φ is related to a Rescorla–Wagner-style learning rate α (*65*), by $\alpha = 1 - \varphi$. A high value of φ means that stimuli keep a high fraction of their previous value (slow learning from reinforcement).

When ρ is high, then "well-known" actions (with high n) are updated relatively little by reinforcement, by virtue of the terms involving n, while reinforcement has a proportionately larger effect on novel actions (with low n). When $\rho = 0$, n is always 1 and the value update rule reduces to Rescorla–Wagner updating [see e.g. (*67*) for full derivation].

Choice is governed by a softmax process with inverse temperature β [*(11)*, equation 5]:

$$P(c_{t+1} = i) = \frac{e^{\beta Q(c=i,t+1)}}{\sum_j \; e^{\beta Q(c=j,t+1)}}$$

where, in this instance, the action value $Q_{c,t}$ is simply the stimulus value $v_{c,t}$.

In our study, we applied the constraint $\beta > 0$ (since negative values of β imply that the preferred choice is less likely to be chosen than others, contrary to the definition of preference). Values of n began at 0; v began at 0.5 (for consistency with other models in this study). We updated n only for stimuli that were chosen. We used $\lambda = 1$ for reward and $\lambda = 0$ for nonreward.

S15. Win-stay, lose-shift model

As another simple control, we ran a standalone model with two free parameters, p(stay | win) and p(shift | loss). Simulated subjects chose at random for their first trial (or for a novel discrimination) and thereafter moved between stimuli in a Markov chain governed by the outcome of the preceding trial and these two transition probabilities.

S16. Resulting models

The resulting models that were compared are shown in **Supplementary Table 1**.

S17. Priors

Priors are shown in **Supplementary Table 2**.

Group means were sampled from the prior distributions shown. The subject-specific component for each parameter was an unchanging property of the subject, drawn from a normal distribution $N(0, \sigma)$ having mean 0 and an (intersubject) standard deviation σ that was estimated (with priors for σ as shown). Final per-subject parameter values were constrained (clipped) to the possible range if applicable.

We note that Ahn et al., via the hBayesDM package (*69*, *73*), use highly standardized priors. Using the notation of μ group mean, σ standard deviation, r raw (internally calculated) per-subject value, x final per-subject value, and $^+$ as constraining to positive, they recommend sampling unconstrained parameters as $\mu \sim N(0, 10)$, $\sigma \sim \text{Cauchy}(0, 5)^+$, $x \sim N(\mu, \sigma)$; positive parameters as $\mu \sim N(0, 1)$, $\sigma \sim \text{Cauchy}(0, 5)^+$, $r \sim N(\mu, \sigma)$, $x = e^r$; parameters in the range [0, 1] as $\mu \sim N(0, 1)$, $\sigma \sim \text{Cauchy}(0, 5)^+$, $r \sim N(\mu, \sigma)$, $x = \Phi(r)$; parameters in the range [0, U] as $\mu \sim N(0, 1)$, $\sigma \sim \text{Cauchy}(0, 5)^+$, $r \sim N(\mu, \sigma)$, $x = U \cdot \Phi(r)$. We attempted this but found that the method often yielded very poor convergence. This may have been because we had small group sizes. Regardless, direct specification of priors, as per **Supplementary Table 2** and following (*67*), led to better convergence, and we used that method throughout. We note future potential to blend these approaches by transforming variables sampled in "normal" space (at group and subject level) via quantile (inverse cumulative distribution) functions for prior distributions of interest.

S18. Fitting

We coded and fitted all models using Stan 2.26.1 via RStan version 2.26.21 (*74*, *75*).

S19. Convergence

We assessed model convergence using the $\hat{R}$ metric. As the AUROC measurement method we used (see below) required that response probabilities for every single trial be "exposed" in the Stan results, we did not require that every single such trial probability converged, but rather that all group mean/SD parameters, all group difference measures, and the AUROC itself converged (as would be the norm). We used a "stringent" criterion of requiring $\hat{R} \leqslant 1.1$, after (*76*). For models that did not converge, we re-attempted using standard mitigations such as increasing Stan's `adapt_delta` setting towards 1 and increasing the `iter` setting (*74*, *75*), but one failed to converge nonetheless (potentially indicating over-parameterization). We summarize the poorly converging model but this was very far from optimal in any case.

S20. Model comparison

We coded all models so as to support bridge sampling (*77*, *78*) and used this as the basis for model comparison. Bridge sampling rewards a good fit and penalizes model complexity, as do other methods, but does so in a theoretically optimal way so as to give P(model | data) given the assumption that the family of models being tested contains the actual model. This method differs from our previous related work in marmosets (*19*, *66*), which used the Bayesian information criterion (BIC); bridge sampling is a more recently available and theoretically optimal method, but they are conceptually similar. We compared models on the basis of their "global" fit (to all subjects, including data from before and after the lesion). We assumed all models being compared were equiprobable *a priori*.

S21. Quantifying fit to behavioural data

While we used bridge sampling (see above) to compare the models, we also included a real-world measure of goodness of fit, the AUROC. This measure quantifies the performance of a binary classifier from 0.5 (random) to 1 (perfect). Values in the range [0, 0.5] are theoretically possible but indicate that a classifier is performing worse than by chance, which would usually indicate a coding error. In our study, all choices were either to the left or right, so a binary classifier was appropriate. We measured performance of the models in predicting the subjects' actual choices (across all subjects and all trials). The predictor was the model's calculated probability (or equivalently log odds) of choosing the left-hand stimulus, and the dependent variable whether the left-hand stimulus was chosen. In our context, the residual "gap" between a classifier's AUROC and 1 may represent a combination of (a) systematic subject performance not captured by the model (but which in principle might be captured by a better model), and/or (b) random error in the subjects' responding (not capturable even in principle). Previous work has suggested interspecies variation in (b) (*79*).

We illustrated model fits by plotting *p*(correct) by trial for each subject, from behavioural data and the model's prediction, using a smoothing window of 20 trials.

S22. Parameter recovery for winning model

We verified parameter recovery from the winning model. The Bayesian method we used for analysis is computationally intensive, and combinatorial exploration of parameter space was not feasible (*67*). We therefore simulated 5 values per parameter, spanning a range that exceeded the range of posterior group mean values from the actual data set. We set all parameters but one to the central value and varied one parameter at a time, as shown in **Supplementary Table 3**. For each run, we simulated 50 identical virtual subjects in a single virtual group performing a simulated behavioural task (see below), capped to 5,000 trials per subject. We analysed the simulated data exactly as we analysed the real data, except removing the measurement of intersubject variability (since in this situation, intersubject variability was zero). For each parameter, we measured the mean and 95% highest density interval (HDI) of its posterior distribution. Results are presented in **Supplementary Table 3**.

S23. Posterior parameters and lesion effects

We express posterior distributions using highest posterior density intervals (HDIs), the Bayesian "credible interval". We sampled posterior distributions for group differences directly.

S24. Simulation of behavioural data for winning model

We simulated behaviour using the posterior group mean parameters from the winning model, with 100 simulated subjects per group (before versus after lesion) and no simulated intersubject variation (*67*), for comparison to behavioural data. The simulation framework replicated the behavioural task, including the pass criterion. (We capped to 10,000 simulated trials per subject, if applicable.) We analysed win-stay/lose-shift probabilities and errors to criterion.

S25. Supplementary results

S26. Model comparison and winning model

Model comparisons are shown in **Supplementary Table 1**. Model convergence was good for all models except one (which was far from the optimal model).

The winning model, model W, incorportated reinforcement learning via a single learning rate (α_{reinf}) and inverse temperature (β_{reinf}); stimulus stickiness via a gradual learning process (α_{stim}) and inverse temperature (β_{stim}); location (side) stickiness likewise (α_{loc}, β_{loc}); and a mixing parameter blending in "ideal" responding via RPM (m).

Of note, the more complex "gradual stickiness" processes previously used in marmoset-based modelling (*19*, *66*) produced a substantial improvement in fit and penalized fit relative to the simple "one-back" stickiness processes

previously used in human (*67*, *79*) and some rodent (*79*) studies. However, in the present study, addition of RPM also improved the penalized fit, and was therefore part of the winning model.

Relative to the winning model (model W), separate reward/punishment learning rates (model X) did not improve fit. Addition of a side bias parameter (model Y) improved AUROC but not parsimoniously, as judged by bridge sampling. Addition of a global reward trace *(7)* (Model Z) did not improve fit. As a technicality, use of RPM via a mixing parameter (m) was broadly similar to using RPM via an inverse temperature method (β_{RPM}) but the mixing parameter method proved slightly superior.

S27. Parameter recovery for winning model

Parameter recovery for the winning model was excellent (**Supplementary Table 3**), with 94.6% of the true parameters being within the 95% HDIs of the recovered parameters (versus an expectation, by definition, of 95%). The range of variation tested for each parameter exceeded the range of posterior group means from marmoset data in both the control and lesion conditions; thus, an appropriate behavioural range was tested for each parameter.

S28. Simulation of behavioural data for winning model

Trial-by-trial behaviour is shown in **Figure S5** for marmosets, before and after lesions, along with the corresponding fits from the winning model.

Conventional behavioural summaries of data simulated from the winning model are shown in **Figure 5B–C** (main manuscript), including errors per discrimination and a "win–stay, lose–shift" analysis, as for marmoset behavioural data (**Figure 4F–G**).

S29. Posterior parameters and lesion effects for winning model

Posterior parameters for all effects (and the overall AUROC) are shown in **Figure S6**. The effects of interest, namely the alterations in group mean parameter values, are shown in **Figure 5A** (main manuscript).

The effects that were credible at the 95% HDI level were a reduction, following the lesion, in the "strength" (inverse temperature parameter) of both stimulus stickiness and location stickiness, and a reduction in the RPM mixing parameter.

S30. Supplementary Tables

Supplementary Table 1. Summary of computational models tested. *, Winning model; †, equivalent to the winning model of *(3, 5)* plus β_{reinf}; ¶, winning model of *(6)*; ‡, poor convergence despite standard mitigations; AUROC, posterior mean area under the receiver operating curve; EWA, experienced-weighted attraction, RPM, randomized probability matching. Values are shown to 4 significant figures. The tree diagram shows relationships between models; this is only one of many such possible trees. The textual notation "mix_rpm1_other0" represents the binary RPM mixing variable *m* described in the **Supplementary Methods**.

**Model**		**Log marginal likelihood**	**Max R-hat for parameters of interest**	**Model rank**	**Log posterior probability of model (via bridge sampling)**	**Mean AUROC**	**AUROC rank**	**Comment**
•	A. RPM_odds/β_RPM	-5959	1.048	27	-599.0	0.7166	27	
•	B. Bayesian discriminations/update unchosen/p_choose = p_correct (p_rc)	-5896	1.026	26	-535.2	0.7227	26	
•	C. p_stay_given_win, p_shift_given_loss (win-stay/lose-shift model)	-6407	1.005	28	-1046.	0.6194	28	
•	D. φ, ρ, β [EWA model]	-5839	1.042	23	-478.8	0.7313	22	
•	E. side_bias	-6518	1.004	29	-1158.	0.5633	29	
•┐	F. a_reinf, β_reinf	-5880	1.045	25	-519.8	0.7237	25	
├•┐	G. a_rew, a_pun, β_reinf	-5824	1.029	22	-463.3	0.7309	23	
│ └•	H. a_rew, a_pun, β_reinf; side_bias	-5712	1.015	13	-351.6	0.7438	16	
├•	I. a_reinf, β_reinf; lapse (ξ)	-5870	1.006	24	-510.0	0.7247	24	
├•┐	J. a_reinf, β_reinf; side_bias	-5769	1.009	17	-408.2	0.7362	21	
│ ├•	K. a_reinf, β_reinf; side_bias; β_loc (one-back)	-5718	1.004	14	-357.2	0.7475	15	
│ └•	L. a_reinf, β_reinf; side_bias; β_stim (one-back)	-5784	1.005	19	-423.2	0.7362	20	
├•┐	M. a_reinf, β_reinf; β_loc (one-back)	-5795	1.004	21	-434.2	0.7401	19	
│ └•┐	N. a_reinf, β_reinf; β_stim (one-back); β_loc (one-back)	-5788	1.005	20	-427.1	0.7410	18	
│ ├•	O. a_reinf, β_reinf; side_bias; β_stim (one-back); β_loc (one-back)	-5708	1.024	12	-347.4	0.7481	14	
│ ├•	P. a_reinf, β_reinf; β_stim (one-back); β_loc (one-back); RPM/mix_rpm1_other0	-5780	1.799 ‡	18	-419.2	0.7425	17	
│ └•┐	Q. a_rew, a_pun, β_reinf; β_stim (one-back); β_loc (one-back)	-5722	1.027	15	-361.2	0.7492	13	¶
│ ├•┐	R. a_rew, a_pun, β_reinf; side_bias; β_stim (one-back); β_loc (one-back)	-5643	1.044	10	-283.0	0.7559	11	
│ │ └•	S. a_rew, a_pun, β_reinf; side_bias; β_stim (one-back); β_loc (one-back); RPM/mix_rpm1_other0	-5647	1.035	11	-287.0	0.7563	10	
│ └•	T. a_rew, a_pun, β_reinf; β_stim (one-back); β_loc (one-back); RPM/mix_rpm1_other0	-5725	1.024	16	-364.8	0.7495	12	
└•┐	U. a_reinf, β_reinf; a_stim, β_stim; a_loc, β_loc	-5368	1.006	4	-7.126	0.7824	5	†
├•	V. a_reinf, β_reinf; side_bias; a_stim, β_stim; a_loc, β_loc	-5371	1.002	6	-10.69	0.7828	4	
├•┐	**W. a_reinf, β_reinf; a_stim, β_stim; a_loc, β_loc; RPM/mix_rpm1_other0 (*)**	-5361	1.031	1	-0.07377	0.7832	2	*** Winner**
│ ├•	X. a_rew, a_pun, β_reinf; a_stim, β_stim; a_loc, β_loc; RPM/mix_rpm1_other0	-5364	1.040	2	-3.346	0.7823	9	
│ └•	Y. a_reinf, β_reinf; side_bias; a_stim, β_stim; a_loc, β_loc; RPM/mix_rpm1_other0	-5364	1.022	3	-3.361	0.7835	1	Best AUROC
│ └•	Z. a_reinf, β_reinf; a_stim, β_stim; a_loc, β_loc; a_R, w_R (global)	-5368	1.006	5	-7.948	0.7824	7	
└•┐	a. a_reinf, β_reinf; a_stim, β_stim; a_loc, β_loc; RPM/β_RPM	-5387	1.052	8	-26.41	0.7824	6	
├•	b. a_rew, a_pun, β_reinf; a_stim, β_stim; a_loc, β_loc; RPM/β_RPM	-5389	1.036	9	-28.17	0.7824	8	
└•	c. a_reinf, β_reinf; side_bias; a_stim, β_stim; a_loc, β_loc; RPM/β_RPM	-5387	1.020	7	-26.10	0.7828	3	

Supplementary Table 2. Priors for computational models. SD, standard deviation; $[]^+$, constrained positive.

Quantity	Prior	Sources
**Parameter group means**		
Parameters constrained [0, 1], including learning rates (α_{reinf}, α_{rew}, α_{pun}, α_{stim}, α_{loc}); forgetting rates (δ, λ); lapse to random choice (ξ); two-model mixing parameters; raw probabilities; EWA model decay parameters (ρ, φ).	Beta(1.2, 1.2)	(*67*, *72*)
Softmax inverse temperatures, β, constrained [0, ∞).	Gamma(α=4.82, β=0.88)	(*67*, *80*)
Side bias, constrained [–1, 1]; global reward weight w_{R}, constrained [–1, 1]; probabilities constrained [0.5, 1].	Sampled raw as for parameters constrained [0, 1], then transformed linearly to target range.	–
**Intersubject variability in parameters**		
Intersubject SD for group means constrained [0, 1].	$[\text{Normal}(0, 0.05)]^+$	(*67*)
Intersubject SD for softmax inverse temperatures.	$[\text{Normal}(0, 1)]^+$	(*67*)

Supplementary Table 3. Parameter recovery for the winning model from simulated data (see **Supplementary Methods**). Virtual subjects (n = 50) were simulated for each row, using the "true" parameters shown, and the parameters estimated (recovered) from the resulting data via a hierarchical Bayesian model as described in the text. Bold highlights parameter variation (which extended beyond the range of the posterior group means from the control/lesion groups) and italics the group with all-central values. Recovered parameters are shown in the format "μ [a, b] (R=$\hat{R}$)" where μ is the posterior mean, [a, b] is the 95% HDI, and $\hat{R}$ is the potential scale factor reduction measure of convergence. † indicates values for which the parameters were not correctly recovered as judged by the 95% HDI (occurring in 11 out of 203 tests, or 5.4%, versus an expectation of 5% for 95% HDIs).

**Process**	**RL**				**Stimulus stickiness**				**Side (location) stickiness**				**RPM**	
**Param.**	α_{reinf}		β_{reinf}		α_{stim}		β_{stim}		α_{loc}		β_{loc}		m	
**Sim.#**	**True**	**Recovered**	**True**	**Recovered**	**True**	**Recovered**	**True**	**Recovered**	**True**	**Recovered**	**True**	**Recovered**	**True**	**Recovered**
1	**0.01**	0.006 [0.002, 0.011] (R=1.002)	2	3.355 [1.550, 5.879] (R=1.002)	0.02	0.019 [0.017, 0.021] (R=1.001)	3	2.989 [2.814, 3.171] (R=1.003)	0.1	0.100 [0.094, 0.106] (R=1.000)	2	2.036 [1.963, 2.107] (R=1.001)	0.1	0.107 [0.089, 0.125] (R=1.000)
2	**0.05**	0.056 [0.042, 0.071] (R=1.000)	2	1.835 [1.488, 2.193] (R=1.001)	0.02	0.020 [0.017, 0.023] (R=1.001)	3	3.049 [2.867, 3.240] (R=1.000)	0.1	0.102 [0.095, 0.109] (R=1.001)	2	1.985 [1.893, 2.082] (R=1.001)	0.1	0.103 [0.076, 0.130] (R=1.001)
3	*0.1*	*0.098 [0.082, 0.116] (R=1.000)*	*2*	*2.128 [1.848, 2.417] (R=1.000)*	*0.02*	*0.019 [0.016, 0.022] (R=1.001)*	*3*	*2.987 [2.796, 3.176] (R=1.001)*	*0.1*	*0.107 [0.099, 0.116] (R=1.001)*	*2*	*1.834 [1.740, 1.935] (R=1.002) †*	*0.1*	*0.081 [0.049, 0.111] (R=1.000)*
4	**0.2**	0.175 [0.155, 0.199] (R=1.002) †	2	2.163 [1.961, 2.381] (R=1.002)	0.02	0.020 [0.017, 0.023] (R=1.001)	3	2.983 [2.801, 3.168] (R=1.001)	0.1	0.104 [0.096, 0.112] (R=1.001)	2	1.909 [1.803, 2.006] (R=1.000)	0.1	0.067 [0.037, 0.097] (R=1.002) †
5	**0.3**	0.309 [0.280, 0.336] (R=1.002)	2	1.944 [1.807, 2.087] (R=1.002)	0.02	0.023 [0.020, 0.026] (R=1.002) †	3	2.852 [2.692, 3.012] (R=1.001)	0.1	0.108 [0.099, 0.117] (R=1.001)	2	1.946 [1.840, 2.049] (R=1.000)	0.1	0.080 [0.052, 0.108] (R=1.001)
6	0.1	0.096 [0.082, 0.110] (R=1.001)	2	2.080 [1.861, 2.291] (R=1.001)	0.01	0.009 [0.007, 0.010] (R=1.001)	3	3.271 [2.935, 3.632] (R=1.001)	0.1	0.103 [0.096, 0.109] (R=1.000)	2	1.995 [1.913, 2.076] (R=1.000)	0.1	0.095 [0.068, 0.122] (R=1.001)
7	0.1	0.105 [0.089, 0.121] (R=1.000)	2	2.071 [1.839, 2.323] (R=1.000)	0.02	0.014 [0.011, 0.017] (R=1.001)	3	3.048 [2.807, 3.305] (R=1.001)	0.1	0.106 [0.098, 0.114] (R=1.001)	2	1.899 [1.807, 1.993] (R=1.001) †	0.1	0.091 [0.061, 0.121] (R=1.001)
8	0.1	0.100 [0.082, 0.119] (R=1.001)	2	2.048 [1.743, 2.355] (R=1.001)	0.03	0.026 [0.023, 0.030] (R=1.001)	3	2.918 [2.757, 3.078] (R=1.001)	0.1	0.102 [0.094, 0.111] (R=0.999)	2	1.918 [1.811, 2.030] (R=1.000)	0.1	0.090 [0.058, 0.123] (R=1.001)
9	0.1	0.102 [0.078, 0.126] (R=1.001)	2	1.901 [1.558, 2.244] (R=1.001)	0.03	0.034 [0.030, 0.038] (R=1.001) †	3	2.871 [2.715, 3.015] (R=1.001)	0.1	0.094 [0.085, 0.103] (R=1.001)	2	1.979 [1.859, 2.099] (R=1.001)	0.1	0.101 [0.070, 0.131] (R=1.000)
10	0.1	0.101 [0.088, 0.115] (R=1.001)	2	1.985 [1.788, 2.185] (R=1.001)	0.02	0.021 [0.018, 0.024] (R=1.000)	**2**	1.979 [1.876, 2.086] (R=1.001)	0.1	0.101 [0.095, 0.107] (R=1.000)	2	2.007 [1.929, 2.088] (R=1.000)	0.1	0.115 [0.091, 0.140] (R=1.001)
11	0.1	0.102 [0.086, 0.120] (R=1.000)	2	2.033 [1.786, 2.280] (R=1.000)	0.02	0.018 [0.015, 0.021] (R=1.000)	**2.5**	2.566 [2.423, 2.734] (R=1.000)	0.1	0.109 [0.102, 0.117] (R=1.000) †	2	1.920 [1.828, 2.010] (R=1.001)	0.1	0.109 [0.076, 0.139] (R=1.000)
12	0.1	0.093 [0.075, 0.112] (R=1.001)	2	2.159 [1.816, 2.488] (R=1.000)	0.02	0.020 [0.017, 0.023] (R=1.000)	**3.5**	3.441 [3.202, 3.694] (R=1.000)	0.1	0.099 [0.090, 0.107] (R=1.000)	2	1.953 [1.838, 2.066] (R=1.000)	0.1	0.103 [0.072, 0.136] (R=1.000)
13	0.1	0.101 [0.079, 0.121] (R=1.000)	2	2.019 [1.722, 2.335] (R=1.000)	0.02	0.018 [0.016, 0.021] (R=1.001)	**4**	4.183 [3.909, 4.467] (R=1.001)	0.1	0.094 [0.085, 0.103] (R=1.001)	2	1.991 [1.873, 2.109] (R=1.001)	0.1	0.114 [0.092, 0.136] (R=1.001)
14	0.1	0.106 [0.086, 0.127] (R=1.001)	2	1.979 [1.692, 2.281] (R=1.000)	0.02	0.021 [0.018, 0.024] (R=1.000)	3	2.953 [2.773, 3.141] (R=1.000)	**0.03**	0.023 [0.019, 0.027] (R=1.001)	2	1.961 [1.784, 2.162] (R=1.001)	0.1	0.086 [0.051, 0.115] (R=1.000)
15	0.1	0.096 [0.078, 0.114] (R=1.001)	2	2.130 [1.833, 2.429] (R=1.001)	0.02	0.020 [0.018, 0.023] (R=1.000)	3	2.942 [2.777, 3.106] (R=1.000)	**0.05**	0.050 [0.045, 0.056] (R=1.001)	2	1.917 [1.782, 2.038] (R=1.001)	0.1	0.077 [0.049, 0.106] (R=1.001)
16	0.1	0.083 [0.069, 0.096] (R=1.002) †	2	2.429 [2.146, 2.752] (R=1.002) †	0.02	0.018 [0.015, 0.021] (R=1.001)	3	2.948 [2.737, 3.151] (R=1.001)	**0.15**	0.156 [0.146, 0.166] (R=1.000)	2	1.905 [1.820, 1.993] (R=1.001) †	0.1	0.078 [0.049, 0.108] (R=1.002)
17	0.1	0.097 [0.082, 0.113] (R=1.000)	2	2.240 [1.981, 2.514] (R=1.001)	0.02	0.018 [0.016, 0.021] (R=1.001)	3	3.041 [2.865, 3.226] (R=1.001)	**0.2**	0.198 [0.188, 0.208] (R=1.000)	2	1.967 [1.885, 2.053] (R=1.000)	0.1	0.087 [0.057, 0.114] (R=1.000)
18	0.1	0.093 [0.074, 0.113] (R=1.001)	2	1.981 [1.642, 2.305] (R=1.001)	0.02	0.021 [0.018, 0.025] (R=1.001)	3	2.905 [2.708, 3.094] (R=1.000)	0.1	0.108 [0.087, 0.128] (R=1.000)	**1**	0.970 [0.848, 1.105] (R=1.001)	0.1	0.108 [0.072, 0.145] (R=1.000)
19	0.1	0.092 [0.075, 0.109] (R=1.000)	2	2.145 [1.833, 2.471] (R=1.000)	0.02	0.021 [0.018, 0.024] (R=1.002)	3	2.907 [2.726, 3.094] (R=1.002)	0.1	0.103 [0.091, 0.114] (R=1.001)	**1.5**	1.431 [1.318, 1.539] (R=1.000)	0.1	0.093 [0.060, 0.127] (R=1.000)

20	0.1	0.105 [0.089, 0.123]<br>(R=1.002)	2	2.008 [1.771, 2.243]<br>(R=1.001)	0.02	0.019 [0.017, 0.022]<br>(R=1.001)	3	3.058 [2.872, 3.253]<br>(R=1.001)	0.1	0.101 [0.096, 0.107]<br>(R=1.000)	**2.5**	2.427 [2.333, 2.520]<br>(R=1.000)	0.1	0.089 [0.065, 0.115]<br>(R=1.001)
21	0.1	0.112 [0.095, 0.129]<br>(R=1.001)	2	1.928 [1.728, 2.125]<br>(R=1.000)	0.02	0.022 [0.019, 0.025]<br>(R=1.000)	3	2.917 [2.762, 3.078]<br>(R=1.000)	0.1	0.103 [0.098, 0.107]<br>(R=1.001)	**3**	3.017 [2.928, 3.105]<br>(R=1.001)	0.1	0.101 [0.084, 0.118]<br>(R=1.000)
22	0.1	0.102 [0.088, 0.119]<br>(R=1.000)	2	1.978 [1.772, 2.184]<br>(R=1.001)	0.02	0.019 [0.017, 0.021]<br>(R=1.001)	3	3.074 [2.918, 3.226]<br>(R=1.001)	0.1	0.105 [0.098, 0.112]<br>(R=1.001)	2	1.966 [1.894, 2.041]<br>(R=1.002)	**0.02**	0.017 [0.001, 0.034]<br>(R=1.000)
23	0.1	0.092 [0.078, 0.108]<br>(R=1.000)	2	2.140 [1.882, 2.410]<br>(R=1.000)	0.02	0.020 [0.017, 0.023]<br>(R=1.001)	3	2.889 [2.715, 3.070]<br>(R=1.001)	0.1	0.103 [0.096, 0.111]<br>(R=1.001)	2	1.985 [1.896, 2.075]<br>(R=1.000)	**0.05**	0.056 [0.029, 0.083]<br>(R=1.000)
24	0.1	0.093 [0.071, 0.114]<br>(R=1.001)	2	2.118 [1.753, 2.518]<br>(R=1.000)	0.02	0.020 [0.017, 0.024]<br>(R=1.001)	3	2.954 [2.747, 3.165]<br>(R=1.002)	0.1	0.108 [0.099, 0.118]<br>(R=1.000)	2	1.893 [1.769, 2.014]<br>(R=1.000)	**0.2**	0.191 [0.154, 0.226]<br>(R=1.001)
25	0.1	0.098 [0.072, 0.125]<br>(R=1.001)	2	1.998 [1.558, 2.431]<br>(R=1.001)	0.02	0.019 [0.015, 0.022]<br>(R=1.001)	3	3.163 [2.846, 3.472]<br>(R=1.001)	0.1	0.102 [0.092, 0.114]<br>(R=1.000)	2	1.975 [1.814, 2.132]<br>(R=1.001)	**0.3**	0.288 [0.248, 0.328]<br>(R=1.000)
26	0.1	0.109 [0.084, 0.135]<br>(R=1.000)	**1**	1.058 [0.882, 1.223]<br>(R=1.001)	0.02	0.020 [0.017, 0.022]<br>(R=1.001)	3	2.959 [2.802, 3.119]<br>(R=1.000)	0.1	0.106 [0.100, 0.113]<br>(R=1.000)	2	1.937 [1.859, 2.014]<br>(R=1.002)	0.1	0.099 [0.076, 0.122]<br>(R=1.001)
27	0.1	0.109 [0.089, 0.132]<br>(R=1.001)	**1.5**	1.424 [1.230, 1.647]<br>(R=1.001)	0.02	0.019 [0.017, 0.022]<br>(R=1.001)	3	3.058 [2.868, 3.245]<br>(R=1.001)	0.1	0.105 [0.098, 0.112]<br>(R=1.000)	2	1.986 [1.893, 2.074]<br>(R=1.001)	0.1	0.112 [0.088, 0.139]<br>(R=1.001)
28	0.1	0.102 [0.086, 0.120]<br>(R=1.001)	**2.5**	2.566 [2.249, 2.921]<br>(R=1.001)	0.02	0.019 [0.017, 0.022]<br>(R=1.001)	3	2.980 [2.795, 3.173]<br>(R=1.001)	0.1	0.104 [0.095, 0.113]<br>(R=1.000)	2	1.849 [1.737, 1.954]<br>(R=1.001) †	0.1	0.083 [0.049, 0.117]<br>(R=1.001)
29	0.1	0.100 [0.084, 0.115]<br>(R=1.000)	**3**	2.982 [2.612, 3.374]<br>(R=1.000)	0.02	0.019 [0.016, 0.023]<br>(R=1.000)	3	2.991 [2.777, 3.215]<br>(R=1.000)	0.1	0.099 [0.090, 0.109]<br>(R=1.001)	2	1.890 [1.764, 2.021]<br>(R=1.000)	0.1	0.097 [0.053, 0.139]<br>(R=1.000)

Supplementary Table 4

Ratios of k_3^s between the target region and its neighbours. As k3s is related to the time-activity curve (TAC) shape, partial volume error (aka spillover) can indirectly affect k3s by changing the time-activity curve shape through weighted local-averaging of TACs due to the limited spatial resolution. We therefore examined the ratio of k3s between the target regions (i.e. caudate tail and globus pallidus) and those of their neighbouring regions (see the table below). For ratios less than one, k3s in the target region will be overestimated due to spillover of higher k3s TACs from the neighbouring regions, whereas a ratio exceeding one will lead to underestimation of the k3s in the target region due to spillover of lower k3s TACs from the neighbouring regions. The degree of under/overestimation will increase with increasing deviation of the k3s ratio from unity. In the table below it can be seen that for all target/neighbour combinations the k3s ratio is higher for the post-degradation scan than the pre-degradation scan. For the globus pallidus, pre-degradation scan k3s will be overestimated and post-degradation scan k3s will be underestimated, while for caudate tail the degree of overestimation (if at all) will be higher for the pre-degradation scan. Hence, the effects of spillover will be lead to an underestimation of the increase in k3s seen in the target regions for the post-degradation scan compared to the pre-degradation scan.

Globus Pallidus				
	EGP/Putamen	EGP/aHypo	IGP/Putamen	IGP/aHypo
Pre	0.69 ± 0.1	0.90 ± 0.34	0.38 ± 0.22	0.58 ± 0.56
Post	1.09 ± 0.60	2.29 ± 0.97	1.12 ± 0.68	2.54 ±1.51
Caudate Tail				
	Caud Tail/ Amyg Ce	Caud Tail/Putamen	Caud Tail/ aHipp	Caud Tail/ Amyg Lat
Pre	0.45 ± 0.32	0.45 ± 0.37	1.14 ± 0.74	1.63 ± 1.2
post	0.76 ± 0.29	0.71 ± 0.22	2.49 ± 1.33	2.38 ± 0.38

Supplementary Table 5.

Pre-degradation and post-degradation regional striatal values for k_3^s, a metric of the rate [^{18}F]-DOPA, and the percentage k_3^s change in each area. * P<0.05.

	Pre degradation				Post degradation				% change
**Brain Region**	**Animal 11**	**Animal 12**	**Animal 13**	**Mean**	**Animal 11**	**Animal 12**	**Animal 13**	**Mean**	
**Dorsolateral Caudate**	0.004583	0.005151	0.006508	0.005414 ± 0.000571	0.005186	0.002967	0.008819	0.005657 ± 0.001706	104.49
**Ventromedial Caudate**	0.006112	0.006382	0.005261	0.005918 ± 0.000338	0.005482	0.004804	0.008052	0.006112 ± 0.000989	103.28
**Caudate Body**	0.002543	0.003255	0.001373	0.00239 ± 0.000549	0.005082	0.004084	-0.00108	0.002694 ± 0.001911	112.7
**Caudate Tail**	0.002811	0.000431	0.002189	0.00181 ± 0.000713	0.005573	0.002206	0.004267	0.004015 ± 0.00098	221.8*
**Putamen**	0.003367	0.004588	0.005345	0.004433 ± 0.000576	0.005937	0.003103	0.0087	0.005913 ± 0.001616	133.38
**Nucleus Accumbens Core**	0.004657	0.004374	0.005447	0.004826 ± 0.000321	0.00668	0.006385	0.004017	0.005694 ± 0.000843	117.99
**Nucleus Accumbens Shell**	0.003755	0.003932	0.004761	0.004149 ± 0.00031	0.007144	0.005356	0.002262	0.004921± 0.001426	118.59
**Globus Pallidus**	0.00287	0.005602	0.005948	0.004807 ± 0.000973	0.008551	0.011392	0.013233	0.011058 ± 0.001362	230.06*

S31. Supplementary FIGURES

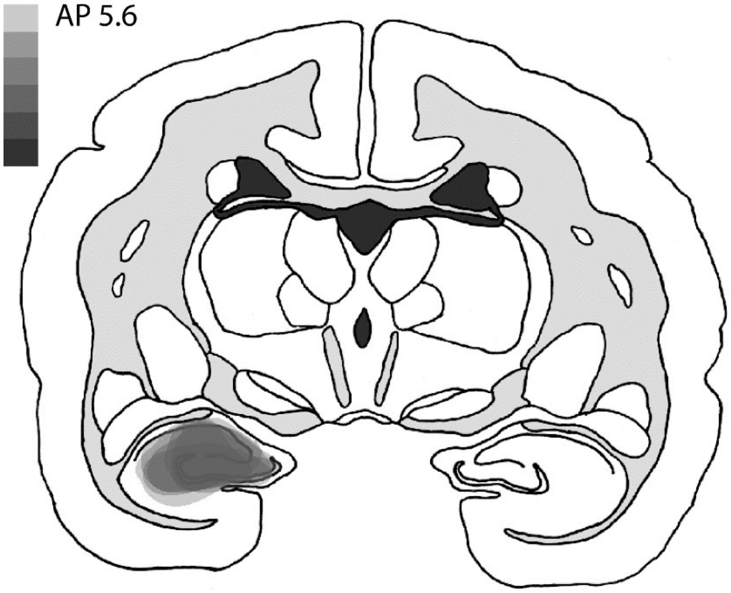

Figure S1. Degradation quantification Schematic diagram at the level of the aHipp at AP 5.6 showing the extent of the unilateral aHipp degradation in the animals that contributed to the quantification of aHipp PNN degradation (in Figure 1). From darkest to lightest, the 6 shades of grey indicate the regions degraded in all 6 monkeys, any 5 monkeys, any 4, any 3, any 2, and 1 monkey.

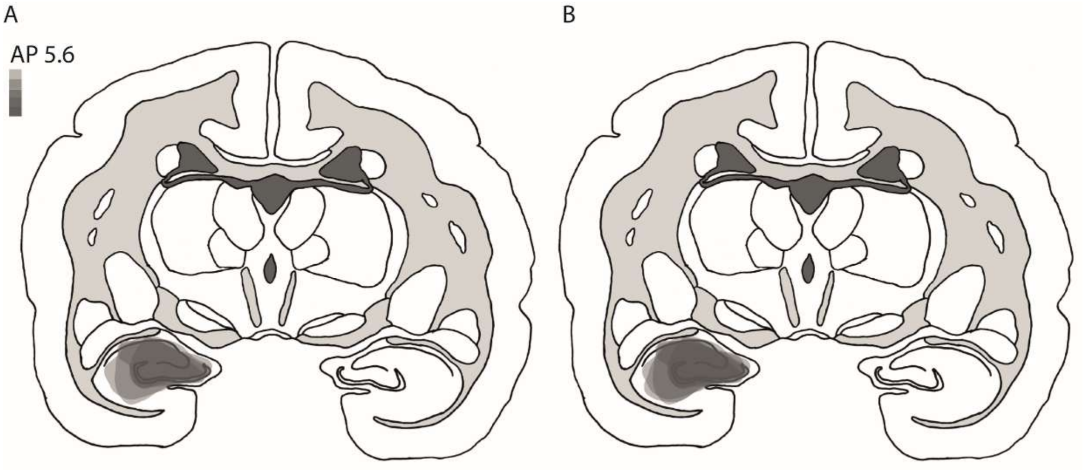

Figure S2. Dialysis histology. Schematic diagram at the level of the aHipp showing the extent of the unilateral aHipp degradation in the animals that received microdialysis in the Acb (**A**; from Figure 2F, n=4) and **B**, the OFC (from Figure 3D, n=5). The 4 animals that received Acb microdialysis also had OFC microdialysis. From darkest to lightest, the 5 shades of grey indicate the regions degraded in all 5 monkeys, any 4 monkeys, any 3, any 2, and 1 monkey.

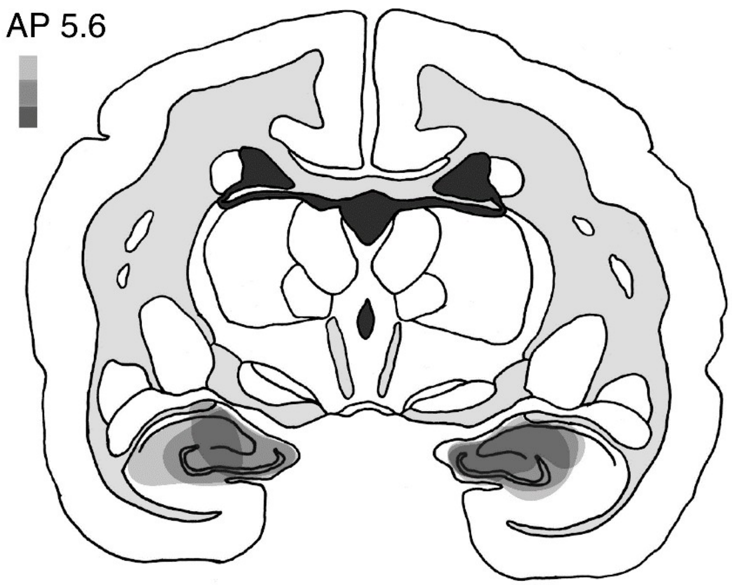

Figure S3. PET histology. Schematic diagram at the level of the aHipp showing the extent of the bilateral aHipp degradation of the PET animals from Figure 2G (n=3). From darkest to lightest, the 3 shades of grey indicate the regions degraded in all 3 monkeys, any 2 monkeys, and 1 monkey.

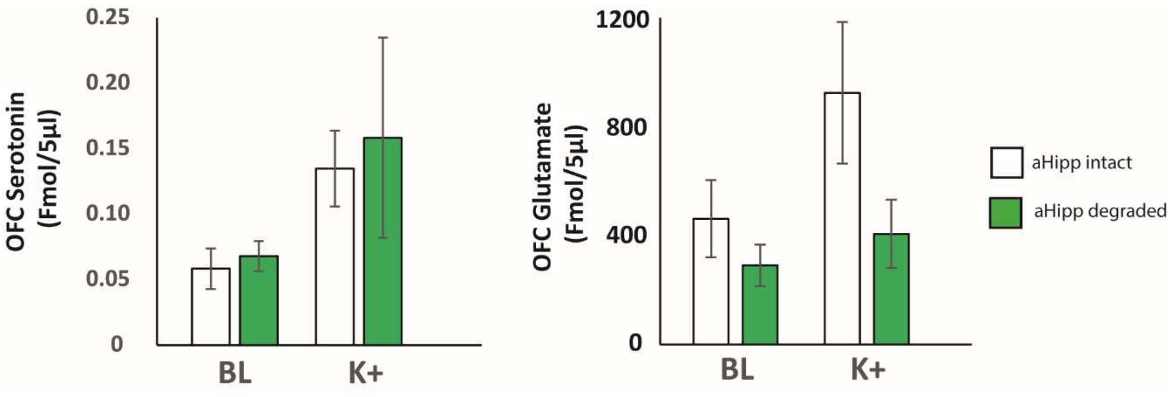

Figure S4. OFC glutamate and 5-HT . Extracellular serotonin and glutamate levels within the OFC at tonic baseline (BL) conditions and phasic conditions (evoked by 75mM K^+; K+). Neither were changed by aHipp PNN degradation (n = 5).

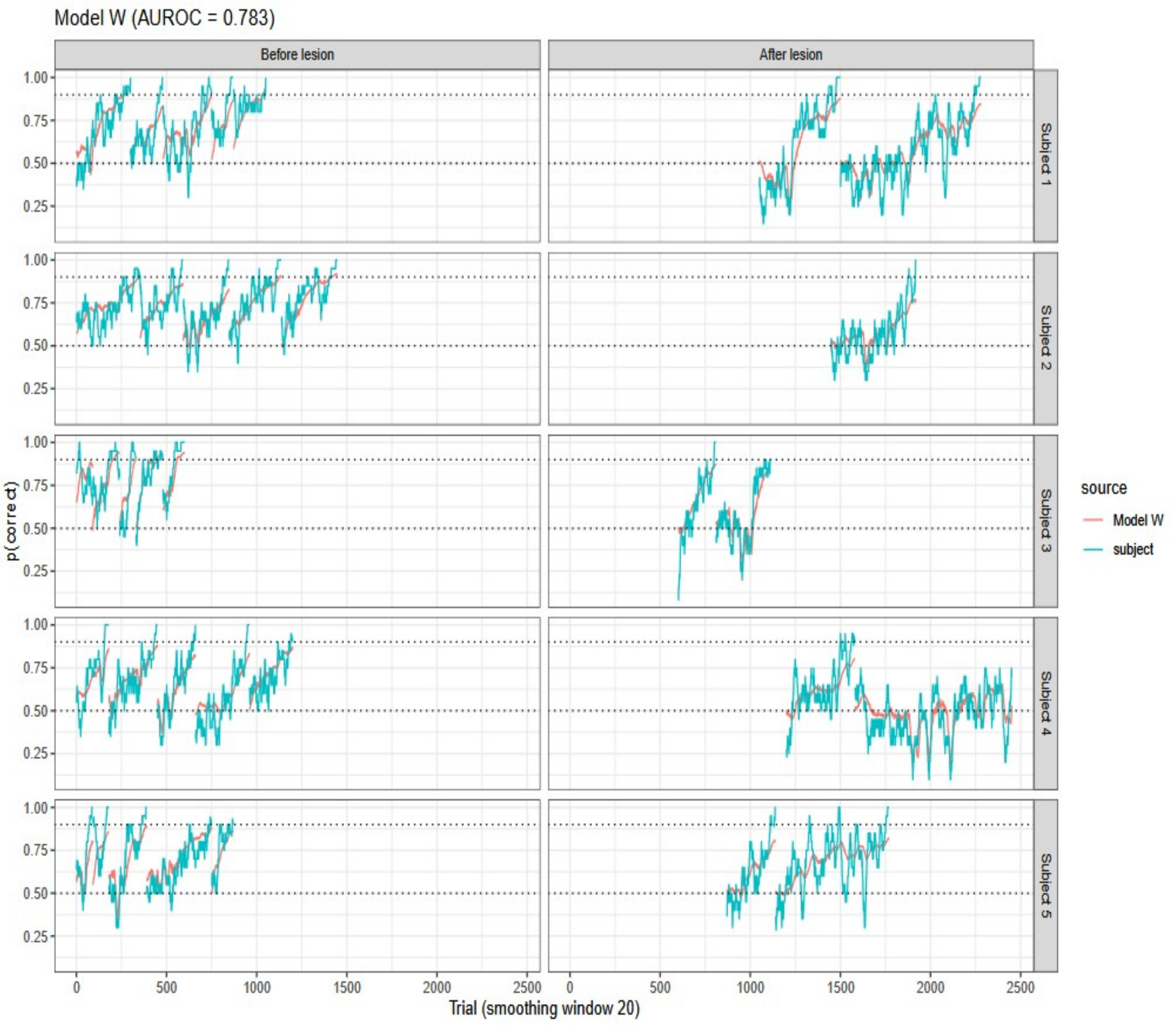

Figure S5. **Trial-by-trial data with the computational model's prediction.** Trial-by-trial probabilities of choosing the correct (optimal) stimulus, from individual marmosets (cyan) and from the winning computational model's fit to those marmosets (red). Both are shown with a smoothing window (see **Supplementary Methods**). Separate lines are shown for each discrimination (stimulus pair). Dotted lines represent chance (p = 0.5) and criterion performance (p = 0.9).

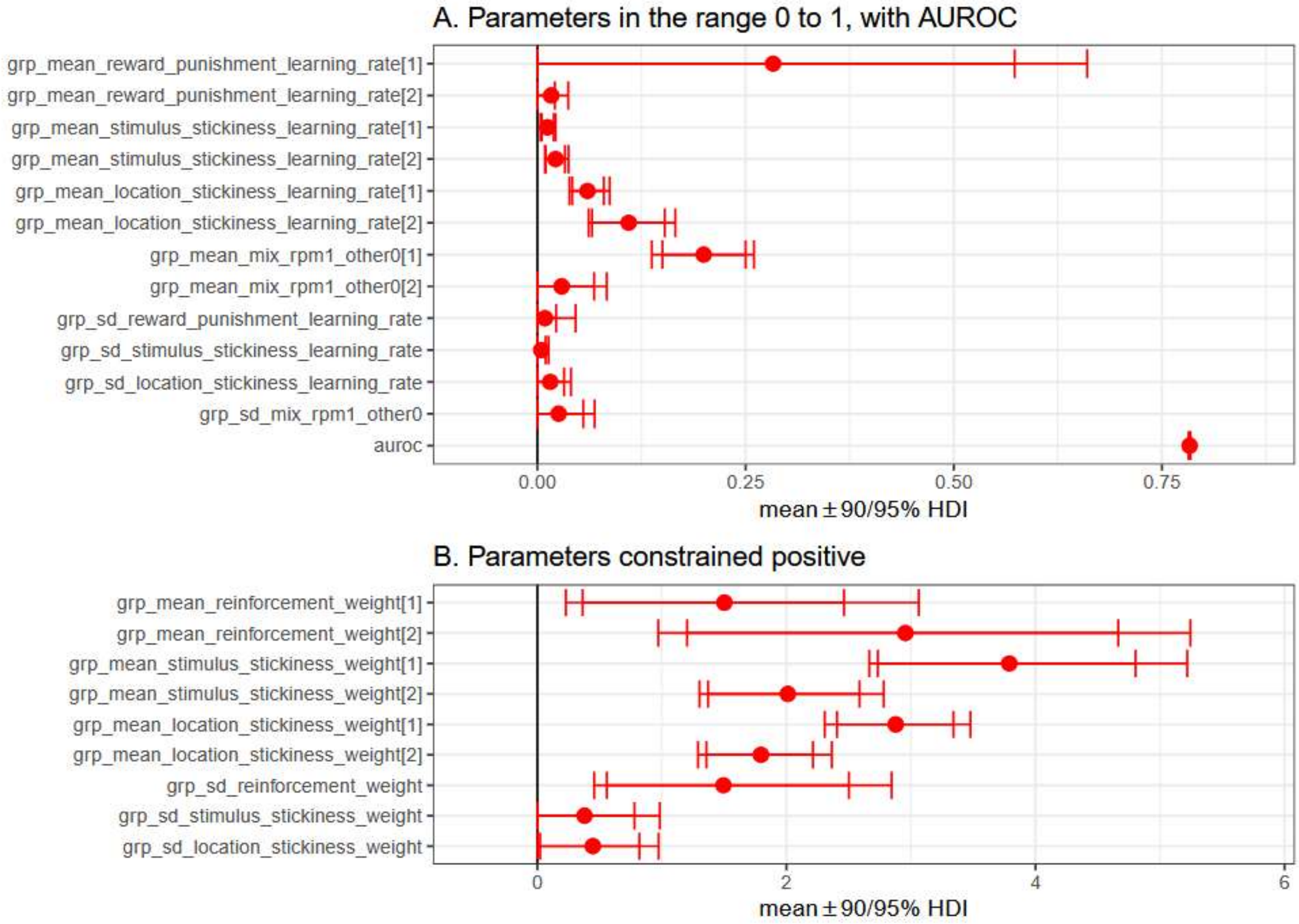

Figure S6. Values of parameters for the winning computational model. Group mean parameters, and the AUROC, are shown as posterior means (dots) with 95% (outer) and 90% (inner) highest posterior density intervals (HDIs). The suffix "[1]" indicates the control condition and "[2]" the lesion condition; "grp", group; "sd", standard deviation; "mix_rpm1_other0" denotes the RPM mixing parameter m. (Red, 0 ∉ 95% HDI; yellow, 0 ∉ 90% HDI; black, 0 ∈ 90% HDI. Note non-normal distributions of some posteriors.) For visual clarity, parameters are divided by range: (**A**) those constrained [0, 1]; (**B**) those constrained [0, ∞). See **Figure 4** for lesion effects.

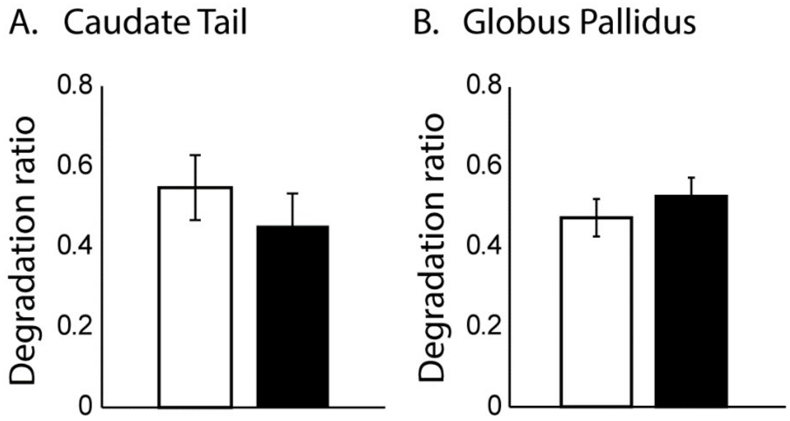

Figure S7. Perineuronal nets were not degraded in the caudate tail or globus pallidus. The ratio of PNN staining in the caudate tail and globus pallidus (as a fraction of all staining) on the side where the hippocampus is degraded (black), is not reduced when compared to the side with an intact hippocampus (white). Caudate, $t_5 = -0.589$, NS; Globus pallidus, $t_5 = 0.572$, NS. Mean ± SEM.

REFERENCES AND NOTES

1. S. Heckers, C. Konradi, GABAergic mechanisms of hippocampal hyperactivity in schizophrenia. *Schizophr Res.* **167**, 4–11 (2015).

2. S. A. Schobel, N. H. Chaudhury, U. A. Khan, B. Paniagua, M. A. Styner, I. Asllani, B. P. Inbar, C. M. Corcoran, J. A. Lieberman, H. Moore, S. A. Small, Imaging patients with psychosis and a mouse model establishes a spreading pattern of hippocampal dysfunction and implicates glutamate as a driver. *Neuron* **78**, 81–93 (2013).

3. S. A. Schobel, N. M. Lewandowski, C. M. Corcoran, H. Moore, T. Brown, D. Malaspina, S. A. Small, Differential targeting of the CA1 subfield of the hippocampal formation by schizophrenia and related psychotic disorders. *Arch. Gen. Psychiatry* **66**, 938–46 (2009).

4. S. J. Kaar, S. Natesan, R. McCutcheon, O. D. Howes, Antipsychotics: Mechanisms underlying clinical response and side-effects and novel treatment approaches based on pathophysiology. *Neuropharmacology* **172**, 107704 (2020).

5. B. Shurman, W. P. Horan, K. H. Nuechterlein, Schizophrenia patients demonstrate a distinctive pattern of decision-making impairment on the Iowa Gambling Task. *Schizophr. Res.* **72**, 215–224 (2005).

6. R. A. McCutcheon, R. S. E. Keefe, P. K. McGuire, Cognitive impairment in schizophrenia: Aetiology, pathophysiology, and treatment. *Mol. Psychiatry* **28**, 1902–1918 (2023).

7. N. Samudra, E. I. Ivleva, N. A. Hubbard, B. Rypma, J. A. Sweeney, B. A. Clementz, M. S. Keshavan, G. D. Pearlson, C. A. Tamminga, Alterations in hippocampal connectivity across the psychosis dimension. *Psychiatry Res. Neuroimaging* **233**, 148–157 (2015).

8. M. B. Moser, E. I. Moser, Functional differentiation in the hippocampus. *Hippocampus* **8**, 608–619 (1998).

9. M. J. Minzenberg, A. R. Laird, S. Thelen, C. S. Carter, D. C. Glahn, Meta-analysis of 41 functional neuroimaging studies of executive function in schizophrenia. *Arch. Gen. Psychiatry* **66**, 811–822. (2009).

10. A. Shah, D. J. Lodge, A loss of hippocampal perineuronal nets produces deficits in dopamine system function: Relevance to the positive symptoms of schizophrenia. *Transl. Psychiatry* **3**, e215 (2013).

11. S. B. Floresco, C. L. Todd, A. A. Grace, Glutamatergic afferents from the hippocampus to the nucleus accumbens regulate activity of ventral tegmental area dopamine neurons. *J. Neurosci.* **21**, 4915–4922 (2001).

12. S. B. Floresco, A. R. West, B. Ash, H. Moorel, A. A. Grace, Afferent modulation of dopamine neuron firing differentially regulates tonic and phasic dopamine transmission. *Nat. Neurosci.* **6**, 968–973 (2003).

13. D. J. Lodge, A. A. Grace, The hippocampus modulates dopamine neuron responsivity by regulating the intensity of phasic neuron activation. *Neuropsychopharmacology* **31**, 1356–1361 (2006).

14. R. McCutcheon, K. Beck, S. Jauhar, O. D. Howes, Defining the locus of dopaminergic dysfunction in schizophrenia: A meta-analysis and test of the mesolimbic hypothesis. *Schizophr. Bull.* **44**, 1301–1311 (2018).

15. P. Fusar-Poli, A. Meyer-Lindenberg, Striatal presynaptic dopamine in schizophrenia, part II: Meta-analysis of [^{18}F/^{11}C]-DOPA PET studies. *Schizophr. Bull.* **39**, 22–32 (2013).

16. S. A. Schobel, M. A. Kelly, C. M. Corcoran, K. Van Heertum, R. Seckinger, R. Goetz, J. Harkavy-Friedman, D. Malaspina, Anterior hippocampal and orbitofrontal cortical structural brain abnormalities in association with cognitive deficits in schizophrenia. *Schizophr. Res.* **114**, 110–118 (2009).

17. Y. Saga, E. Hoshi, L. Tremblay, Roles of multiple globus pallidus territories of monkeys and humans in motivation, cognition and action: An anatomical, physiological and pathophysiological review. *Front. Neuroanat.* **11**, 30 (2017).

18. G. P. Reynolds, Increased concentrations and lateral asymmetry of amygdala dopamine in schizophrenia. *Nature* **305**, 527–529 (1983).

19. H. F. Clarke, R. N. Cardinal, R. Rygula, Y. T. Hong, T. D. Fryer, S. J. Sawiak, V. Ferrari, G. Cockcroft, F. I. Aigbirhio, T. W. Robbins, A. C. Roberts, Orbitofrontal dopamine depletion upregulates caudate dopamine and alters behavior via changes in reinforcement sensitivity. *J. Neurosci.* **34**, 7663–76 (2014).

20. L. F. Reddy, J. A. Waltz, M. F. Green, J. K. Wynn, W. P. Horan, Probabilistic reversal learning in schizophrenia: Stability of deficits and potential causal mechanisms. *Schizophr. Bull.* **42**, 942–951 (2016).

21. C. D. Frith, D. J. Done, Stereotyped responding by schizophrenic patients on a two-choice guessing task. *Psychol. Med.* **13**, 779–786 (1983).

22. J. P. Kesby, D. W. Eyles, J. J. McGrath, J. G. Scott, Dopamine, psychosis and schizophrenia: The widening gap between basic and clinical neuroscience. *Transl. Psychiatry* **8**, 30 (2018).

23. D. M. Staton, P. R. Solomon, Microinjections of d-amphetamine into the nucleus accumbens and caudate-putamen differentially affect stereotypy and locomotion in the rat. *Phys. Psych.* **12**, 159–162 (1984).

24. A. J. J. Pijnenburg, W. M. M. Honig, J. A. M. Van Der Heyden, J. M. Van Rossum, Effects of chemical stimulation of the mesolimbic dopamine system upon locomotor activity. *Eur. J. Pharmacol.* **35**, 45–58 (1976).

25. A. J. J. Pijnenburg, W. M. M. Honig, J. M. Van Rossum, Inhibition of d-amphetamine-induced locomotor activity by injection of haloperidol into the nucleus accumbens of the rat. *Psychopharmacologia* **41**, 175–180 (1975).

26. L. S. Kegeles, A. Abi-Dargham, W. G. Frankle, R. Gil, T. B. Cooper, M. Slifstein, D. R. Hwang, Y. Huang, S. N. Haber, M. Laruelle, Increased synaptic dopamine function in associative regions of the striatum in schizophrenia. *Arch. Gen. Psychiatry* **67**, 231 (2010).

27. G. D. Davis, Caudate lesions and spontaneous locomotion in the monkey. *Neurology* **8**, 135 (1958).

28. D. Grabli, K. Mccairn, E. C. Hirsch, Y. Agid, J. Féger, C. François, L. Tremblay, Behavioural disorders induced by external globus pallidus dysfunction in primates: I. Behavioural study. *Brain* **127**, 2039, 2054, (2004).

29. V. Sgambato-Faure, L. Tremblay, Dopamine and serotonin modulation of motor and non-motor functions of the non-human primate striato-pallidal circuits in normal and pathological states. *J. Neural. Transm.* **125**, 485–500 (2018).

30. B. Costall, R. J. Naylor, J. E. Olley, Stereotypic and anticataleptic activities of amphetamine after intracerebral injections. *Eur. J. Pharmacol.* **18**, 83–94 (1972).

31. A. L. Whone, R. Y. Moore, P. P. Piccini, D. J. Brooks, Plasticity of the nigropallidal pathway in Parkinson's disease. *Ann. Neurol.* **53**, 206–213 (2003).

32. O. Bayar Kapici, Y. Kapici, A. Tekın, M. Şırık, A novel diagnosis method for schizophrenia based on globus pallidus data. *Psychiatry Res. Neuroimaging* **336**, 111732 (2023).

33. H. S. Crofts, J. W. Dalley, P. Collins, J. C. Van Denderen, B. J. Everitt, T. W. Robbins, A. C. Roberts, Differential effects of 6-OHDA lesions of the frontal cortex and caudate nucleus on the ability to acquire an attentional set. *Cereb. Cortex* **11**, 1015–1026 (2001).

34. R. M. Ridley, T. A. Haystead, H. F. Baker, An analysis of visual object reversal learning in the marmoset after amphetamine and haloperidol. *Pharmacol. Biochem. Behav.* **14**, 345–351 (1981).

35. R. Cools, R. A. Barker, B. J. Sahakian, T. W. Robbins, Enhanced or impaired cognitive function in Parkinson's disease as a function of dopaminergic medication and task demands. *Cereb. Cortex* **11**, 1136–1143 (2001).

36. C. A. Seger, The visual corticostriatal loop through the tail of the caudate: Circuitry and function. *Front. Syst. Neurosci.* **7**, 104 (2013).

37. I. Divac, H. E. Rosvold, M. K. Szwarcbart, Behavioral effects of selective ablation of the caudate nucleus. *J. Comp. Physiol. Psychol.* **63**, 184–190 (1967).

38. E. Teng, L. Stefanacci, L. R. Squire, S. M. Zola, Contrasting effects on discrimination learning after hippocampal lesions and conjoint hippocampal-caudate lesions in monkeys. *J. Neurosci.* **20**, 3853–3863 (2000).

39. B. F. Sadacca, A. M. Wikenheiser, G. Schoenbaum, Toward a theoretical role for tonic norepinephrine in the orbitofrontal cortex in facilitating flexible learning. *Neuroscience* **345**, 124–129 (2017).

40. E. Seu, A. Lang, R. J. Rivera, J. D. Jentsch, Inhibition of the norepinephrine transporter improves behavioral flexibility in rats and monkeys. *Psychopharmacology* **202**, 505–519 (2009).

41. G. K. Murray, F. Cheng, L. Clark, J. H. Barnett, A. D. Blackwell, P. C. Fletcher, T. W. Robbins, E. T. Bullmore, P. B. Jones, Reinforcement and reversal learning in first-episode psychosis. *Schizophr. Bull.* **34**, 848–855 (2008).

42. J. C. Cerpa, A. Piccin, M. Dehove, M. Lavigne, E. J. Kremer, M. Wolff, S. L. Parkes, E. Coutureau, Inhibition of noradrenergic signalling in rodent orbitofrontal cortex impairs the updating of goal-directed actions. *eLife* **12** (2023).

43. J. T. Merchant, E. K. Moran, M. J. Strube, D. M. Barch, Correlates of real-world goal-directed behavior in schizophrenia. *Psychol. Med.* **53**, 2409–2417 (2023).

44. R. W. Morris, C. Cyrzon, M. J. Green, M. E. Le Pelley, B. W. Balleine, Impairments in action-outcome learning in schizophrenia. *Transl. Psychiatry* **8**, 54 (2018).

45. D. P. van Kammen, M. Kelley, Dopamine and norepinephrine activity in schizophrenia: An intergrative perspective. *Schizophr. Res.* **4**, 173–191 (1991).

46. A. Breier, O. M. Wolkowitz, A. Roy, W. Z. Potter, D. Pickar, Plasma norepinephrine in chronic schizophrenia. *Am. J. Psychiatry* **147**, 1467–1470 (1990).

47. V. Mäki-Marttunen, O. A. Andreassen, T. Espeseth, The role of norepinephrine in the pathophysiology of schizophrenia. *Neurosci. Biobehav. Rev.* **118**, 298–314 (2020).

48. H. Barbas, G. J. Blatt, Topographically specific hippocampal projections target functionally distinct prefrontal areas in the rhesus monkey. *Hippocampus* **5**, 511–533 (1995).

49. D. Levcik, T. Nekovarova, E. Antosova, A. Stuchlik, D. Klement, The role of the hippocampus in object discrimination based on visual features. *Neurobiol. Learn. Mem.* **155**, 127–135 (2018).

50. A. C. Roberts, D. L. Tomic, C. H. Parkinson, T. A. Roeling, D. J. Cutter, T. W. Robbins, B. J. Everitt, Forebrain connectivity of the prefrontal cortex in the marmoset monkey (Callithrix jacchus): An anterograde and retrograde tract-tracing study. *J. Comp. Neurol.* **502**, 86–112 (2007).

51. C. Romberg, S. Yang, R. Melani, M. R. Andrews, A. E. Horner, M. G. Spillantini, T. J. Bussey, J. W. Fawcett, T. Pizzorusso, L. M. Saksida, Depletion of perineuronal nets enhances recognition memory and long-term depression in the perirhinal cortex. *J. Neurosci.* **33**, 7057–65 (2013).

52. R. Cools, M. J. Frank, S. E. Gibbs, A. Miyakawa, W. Jagust, M. D'Esposito, Striatal dopamine predicts outcome-specific reversal learning and its sensitivity to dopaminergic drug administration. *J. Neurosci.* **29**, 1538–1543 (2009).

53. L. Wahl, R. Chirakal, G. Firnau, E. S. Garnett, C. Nahmias, The distribution and kinetics of [^{18}F]6-Fluoro-3-O-methyl-L-dopa in the human brain. *J. Cereb. Blood Flow Metab.* **14**, 664–670 (1994).

54. M. Defrise, P. E. Kinahan, D. W. Townsend, C. Michel, M. Sibomana, D. F. Newport, Exact and approximate rebinning algorithms for 3-d pet data. *IEEE Trans. Med. Imaging* **16**, 145–158 (1997).

55. H. M. Hudson, R. S. Larkin, Accelerated image reconstruction using ordered subsets of projection data. *IEEE Trans. Med. Imaging* **13**, 601–609 (1994).

56. S. J. Sawiak, Y. Shiba, L. Oikonomidis, C. P. Windle, A. M. Santangelo, H. Grydeland, G. Cockcroft, E. T. Bullmore, A. C. Roberts, Trajectories and milestones of cortical and subcortical development of the marmoset brain from infancy to adulthood. *Cereb. Cortex* **28**, 4440–4453 (2018).

57. C. S. Patlak, R. G. Blasberg, Graphical evaluation of blood-to-brain transfer constants from multiple-time uptake data. *J. Cereb. Blood Flow Metab.* **5**, 584–590 (1985).

58. H. Hoshi, H. Kuwabara, G. Léger, P. Cumming, M. Guttman, A. Gjedde, 6-[^{18}F]fluoro-L-DOPA metabolism in living human brain: A comparison of six analytical methods. *J. Cereb. Blood Flow Metab.* **13**, 57–69 (1993).

59. S. J. Sawiak, N. I. Wood, G. B. Williams, A. J. Morton, T. A. Carpenter, Voxel-based morphometry with templates and validation in a mouse model of Huntington's disease. *Magn. Reson. Imaging* **31**, 1522–1531 (2013).

60. Y. Mikheenko, Y. Shiba, S. Sawiak, K. Braesicke, G. Cockcroft, H. Clarke, A. C. Roberts, Serotonergic, brain volume and attentional correlates of trait anxiety in primates. *Neuropsychopharmacology* **40**, 1395–1404 (2015).

61. J. Ashburner, A fast diffeomorphic image registration algorithm. *Neuroimage* **38**, 95–113 (2007).

62. H. F. Clarke, S. C. Walker, J. W. Dalley, T. W. Robbins, A. C. Roberts, Cognitive inflexibility after prefrontal serotonin depletion is behaviourally and neurochemically specific. *Cereb. Cortex* **17**, 18–27 (2006).

63. C. J. C. H. Watkins, "Learning from delayed rewards," thesis, University of Cambridge (1989).

64. C. J. C. H. Watkins, P. Dayan, Q-learning. *Mach. Learn.* **8**, 279–292 (1992).

65. R. A. Rescorla, A. R. Wagner, "A theory of Pavlovian conditioning: Variations in the effectiveness of reinforcement and non-reinforcement" in *Classical Conditioning II: Current Research and Theory*, A. Black, W. Prokasy, Eds. (Appleton-Century-Crofts, 1972), pp. 64–99.

66. R. Rygula, H. F. H. F. Clarke, R. N. R. N. Cardinal, G. J. G. J. Cockcroft, J. Xia, J. W. J. W. Dalley, T. W. T. W. Robbins, A. C. A. C. Roberts, Role of central serotonin in anticipation of rewarding and punishing outcomes: Effects of selective amygdala or orbitofrontal 5-HT depletion. *Cereb. Cortex* **25**, 3064–3076 (2015).

67. J. W. Kanen, K. D. Ersche, N. A. Fineberg, T. W. Robbins, R. N. Cardinal, Computational modelling reveals contrasting effects on reinforcement learning and cognitive flexibility in stimulant use disorder and obsessive-compulsive disorder: Remediating effects of dopaminergic D2/3 receptor agents. *Psychopharmacology* **236**, 2337–2358 (2019).

68. M. K. Wittmann, E. Fouragnan, D. Folloni, M. C. Klein-Flügge, B. K. H. Chau, M. Khamassi, M. F. S. Rushworth, Global reward state affects learning and activity in raphe nucleus and anterior insula in monkeys. *Nat. Commun.* **11**, 3771 (2020).

69. W.-Y. Ahn, N. Haines, L. Zhang, Revealing neurocomputational mechanisms of reinforcement learning and decision-making with the hBayesDM package. *Comput. Psychiatr.* **1**, 24–57 (2020).

70. L. Scott, A modern Bayesian look at the multi-armed bandit. *Appl. Stoch. Models Bus. Ind.* **26**, 639–658 (2010).

71. C. Camerer, T. H. Ho, Experience-weighted attraction learning in normal form games. *Econometrica* **67**, 827–874 (1999).

72. H. E. den Ouden, N. D. Daw, G. Fernandez, J. A. Elshout, M. Rijpkema, M. Hoogman, B. Franke, R. Cools, Dissociable effects of dopamine and serotonin on reversal learning. *Neuron* **80**, 1090–1100 (2013).

73. R. J. Romeu, N. Haines, W. Y. Ahn, J. R. Busemeyer, J. Vassileva, A computational model of the Cambridge gambling task with applications to substance use disorders. *Drug Alcohol Depend.* **206**, 107711 (2020).

74. Stan Development Team, *Stan Modeling Language User's Guide and Reference Manual. Version 2.21* (Stan Development Team, 2020).

75. Stan Development Team, RStan: the R interface to Stan. R package version 2.21.2 (2020).

76. S. P. Brooks, A. Gelman, General methods for monitoring convergence of iterative simulations. *J. Comput. Graph. Stat.* **7**, 434–455 (1998).

77. Q. F. Gronau, H. Singmann, E. J. Wagenmakers, Bridgesampling: An R package for estimating normalizing constants. *J. Stat. Softw.* **92**, 1–29 (2020).

78. Q. F. Gronau, A. Sarafoglou, D. Matzke, A. Ly, U. Boehm, M. Marsman, D. S. Leslie, J. J. Forster, E. J. Wagenmakers, H. Steingroever, A tutorial on bridge sampling. *J. Math Psychol.* **81**, 80–97 (2017).

79. Q. Luo, J. W. Kanen, A. Bari, N. Skandali, C. Langley, G. M. Knudsen, J. Alsiö, B. U. Phillips, B. J. Sahakian, R. N. Cardinal, T. W. Robbins, Comparable roles for serotonin in rats and humans for computations underlying flexible decision-making. *Neuropsychopharmacology* **49**, 600–608 (2024).

80. S. J. Gershman, Empirical priors for reinforcement learning models. *J. Math Psychol.* **71**, 1–6 (2016).
